# Supplementary material for: CFTR Protects against Mycobacterium abscessus Infection by Fine-Tuning Host Oxidative Defenses
Source: Cell Rep. Author manuscript; Available in PMC 2025 Nov 18. (PMC7618368; doi:10.1016/j.celrep.2019.01.071)
Supplement: Supplementary Material — Supplemental Information includes one table and seven figures and can be found with this article online at https://doi.org/10.1016/j.celrep.2019.01.071. [file EMS209705-supplement-Supplementary_Material.zip › 1-s2.0-S2211124719301007-mmc2.pdf]

Cell Reports

Article

CFTR Protects against *Mycobacterium abscessus* Infection by Fine-Tuning Host Oxidative Defenses

Graphical Abstract

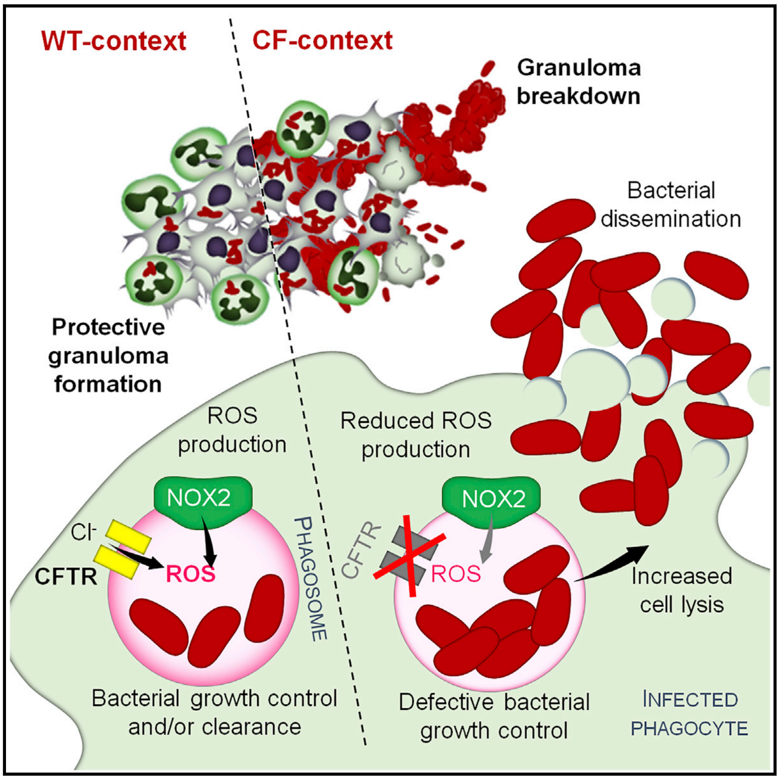

Highlights

- CFTR impairment decreases the phagocyte oxidative defense by limiting ROS production
- CFTR is required for neutrophil recruitment toward forming granulomas
- Granuloma breakdown leads to extracellular mycobacterial expansion
- Dysfunction of CFTR results in hypersusceptibility to *M. abscessus* infection

Authors

Audrey Bernut, Christian Dupont, Nikolay V. Ogryzko, ..., R. Andres Floto, Stephen A. Renshaw, Laurent Kremer

Correspondence

a.bernut@sheffield.ac.uk (A.B.),
laurent.kremer@irim.cnrs.fr (L.K.)

In Brief

Bernut et al. investigate the mechanism by which cystic fibrosis patients are vulnerable to *Mycobacterium abscessus* infection. Using zebrafish, they show that dysfunction of CFTR reduces both macrophage bactericidal activity and neutrophil recruitment to the forming protective granulomas. Together, this leads to hypersusceptibility to *M. abscessus* infection and larval death.

Bernut et al., 2019, Cell Reports *26*, 1828–1840
February 12, 2019 © 2019 The Author(s).
https://doi.org/10.1016/j.celrep.2019.01.071

CellPress

OPEN ACCESS
CellPress

Cell Reports
Article

CFTR Protects against *Mycobacterium abscessus* Infection by Fine-Tuning Host Oxidative Defenses

Audrey Bernut,[1,2,3,*] Christian Dupont,[1] Nikolay V. Ogryzko,[2,3,7] Aymeric Neyret,[1] Jean-Louis Herrmann,[4] R. Andres Floto,[5] Stephen A. Renshaw,[2,3] and Laurent Kremer[1,6,8,*]
[1]CNRS, UMR9004, Institut de Recherche en Infectiologie de Montpellier (IRIM), Université de Montpellier, Montpellier, France
[2]Bateson Centre, University of Sheffield, Sheffield, UK
[3]Department of Infection, Immunity and Cardiovascular Disease, Medical School, University of Sheffield, Sheffield, UK
[4]2I, INSERM, UVSQ, Université Paris-Saclay, Versailles, France
[5]Molecular Immunity Unit, Department of Medicine, University of Cambridge, MRC Laboratory of Molecular Biology, Cambridge, UK
[6]INSERM, IRIM, Montpellier, France
[7]Present address: MRC Centre for Inflammation Research, Queen's Medical Research Institute, University of Edinburgh, Edinburgh, UK
[8]Lead Contact
*Correspondence: a.bernut@sheffield.ac.uk (A.B.), laurent.kremer@irim.cnrs.fr (L.K.)
https://doi.org/10.1016/j.celrep.2019.01.071

SUMMARY

Infection by rapidly growing *Mycobacterium abscessus* is increasingly prevalent in cystic fibrosis (CF), a genetic disease caused by a defective CF transmembrane conductance regulator (CFTR). However, the potential link between a dysfunctional CFTR and vulnerability to *M. abscessus* infection remains unknown. Herein, we exploit a CFTR-depleted zebrafish model, recapitulating CF immuno-pathogenesis, to study the contribution of CFTR in innate immunity against *M. abscessus* infection. Loss of CFTR increases susceptibility to infection through impaired NADPH oxidase-dependent restriction of intracellular growth and reduced neutrophil chemotaxis, which together compromise granuloma formation and integrity. As a consequence, extracellular multiplication of *M. abscessus* expands rapidly, inducing abscess formation and causing lethal infections. Because these phenotypes are not observed with other mycobacteria, our findings highlight the crucial and specific role of CFTR in the immune control of *M. abscessus* by mounting effective oxidative responses.

INTRODUCTION

Cystic fibrosis (CF) is a lethal genetic disorder caused by deleterious mutations in the CF transmembrane conductance regulator (CFTR) protein (Gadsby et al., 2006), resulting in compromised mucociliary clearance, chronic bacterial infections, and subsequent progressive inflammatory lung damage (Donaldson and Boucher, 2003). CF-related lung infections are associated with a specific spectrum of colonizing microorganisms: highly prevalent bacteria such as *Staphylococcus aureus* and *Pseudomonas aeruginosa* (Lyczak et al., 2002) and also emerging bacterial pathogens such as nontuberculous mycobacteria (NTM) (Olivier et al., 2003; Roux et al., 2009). Among the rapidly growing NTM, the *Mycobacterium abscessus* complex (MABSC) represents the most common species found in CF airways (Floto et al., 2016) and is emerging as a major CF pathogen, in part because of indirect person-to-person transmission (Bryant et al., 2013), and progressing into severe pneumonia and accelerated inflammatory lung damage (Esther et al., 2010). Their presence is also a relative contraindication to lung transplantation (Orens et al., 2006). In particular, pulmonary infections with the multidrug-resistant *M. abscessus* subspecies (subsp.) *abscessus* (Mabs) (Nessar et al., 2012) are extremely challenging to treat, requiring aggressive and extended therapies with a high rate of therapeutic failure (DaCosta et al., 2017). To date, Mabs is widely considered to be the most significant rapidly growing NTM in CF, with a worldwide prevalence rate of 5%–20% (Floto et al., 2016).

Mabs exhibits two distinct morphotypes, relying on the presence or absence of surface-associated glycopeptidolipids (GPLs): a smooth (S) high-GPL producer variant and a rough (R) low-GPL producer variant (Howard et al., 2006). Both morphotypes are recovered from the CF airways during infection, but case reports indicate that the R form correlates with exacerbations of pulmonary disease and rapid decline of lung function in the patients (Jönsson et al., 2007; Catherinot et al., 2009). Nevertheless, the specific vulnerability of the CF population to Mabs, the potential link with CFTR dysfunction, and how these mycobacteria contribute to progression of lung disease remain unknown.

Although it is assumed that susceptibility to infections in CF results from defective mucociliary activity, CFTR dysfunction may also alter the inflammatory potential of innate immune cells, contributing to the infectious pathology in this disease. Various hypotheses have attempted to explain the impairment of innate defenses in CF, although these await definitive proof. Numerous mammalian models (Lavelle et al., 2016) have been generated to investigate the role of CFTR dysfunction in innate immunity and hypersusceptibility to infections. However, these models are not suited for direct, real-time imaging of the early processes leading to disease development. Thus, new animal models that approximate the human altered immune phenotype and allow direct visualization of host-pathogen interactions would provide

This is an open access article under the CC BY license (http://creativecommons.org/licenses/by/4.0/).

much needed tools to establish *in vivo* how CFTR regulates innate immunity and controls Mabs infection.

Zebrafish (ZF) larval innate immunity is homologous to that of human (Renshaw and Trede, 2012), and their optical transparency allows non-invasive, real-time monitoring of infection outcomes and host-pathogen interactions. Thus, ZF innate immune cell behavior and function can be visualized at sub-cellular resolution in the whole living animal, allowing dissecting the innate immune response during infectious diseases (Torraca et al., 2014). ZF led to new insights into the virulence of several CF bacteria, especially *P. aeruginosa* (Clatworthy et al., 2009), *S. aureus* (Prajsnar et al., 2008), and *Burkholderia cenocepacia* (Vergunst et al., 2010), and provide a useful model to study the pathophysiology of human Mabs infection (Bernut et al., 2014, 2016a). Importantly, from a structural perspective, ZF CFTR closely resembles the human protein (Zhang and Chen, 2016; Liu et al., 2017), and several reports suggest functional conservation of CFTR between ZF and human (Navis and Bagnat, 2015; Phennicie et al., 2010), making ZF larvae a clinically relevant biological system. Indeed, several CF phenotypes that mirror human CF disease were reported in CFTR-defective ZF, including pancreatitis (Navis and Bagnat, 2015) and increased susceptibility to *P. aeruginosa* (Phennicie et al., 2010).

Herein, using CFTR-depleted ZF larvae as an innovative vertebrate model that recapitulates important aspects of the CF immuno-pathogenesis, we elucidated the role of CFTR in regulation of innate immunity to Mabs infections. We also interrogated the effects of CFTR ablation on host immunity, inflammation, and infection independent of the overlapping infection and inflammation associated with the CF-lung microenvironment. Importantly, our findings emphasize that differential CFTR-dependent ROS production allows the host to adjust inflammatory responses by modulating phagocyte bactericidal functions and their lifespan upon infection, which together ensure the maintenance of a protective granulomatous structure to sequester and control Mabs infection.

RESULTS

Loss of CFTR Function Increases the Severity of *M. abscessus* Infection

To address the role of CFTR in Mabs infection, *cftr* loss-of-function experiments were carried out in ZF using a specific morpholino-modified oligonucleotide (MO) (Figures S1A and S1B). Although *cftr* is expressed and localized to the apical membrane or vesicular compartments of cells (Del Porto et al., 2011) (Figure S1C), *cftr*-MO injection abrogated production of native spliced *cftr* transcripts (Figures S1A and S1B) and altered *cftr* expression (Figure S1D). Embryos injected with *cftr*-MO survived similarly to control-MO injected animals and appeared morphologically similar throughout the observed periods (data not shown). Additionally, to support the knockdown results, we took advantage of the *cftr* ZF mutant (Navis et al., 2013). Upon intravenous infection, both *cftr* morphants and *cftr* mutants displayed hypersusceptibility to R and S Mabs morphotypes, correlating with increased larval mortality (Figure 1A) and higher bacterial burdens, as demonstrated by determination of the fluorescent pixel count (FPC; Figure 1B) and whole-larvae imaging (Figure 1C). The pronounced increase in bacterial loads in CFTR-deficient animals correlates with replicating extracellular bacteria (Figures 1D–1H), translating into larger numbers of larvae with abscesses and with increased number of abscesses per larva in the CNS (Figures 1D and 1E). Mabs abscesses represent a marker of disease severity and uncontrolled infection caused by extracellular replicating mycobacteria that are often associated with cellular debris, tissue destruction, and acute infection in ZF (Bernut et al., 2014). Whereas the S form induces abscesses only rarely in wild-type (WT) fish (Bernut et al., 2014), 30% of Mabs S-infected larvae exhibited abscesses at 3 days post-infection (dpi) in the absence of CFTR (Figure 1D). Electron microscopy (EM) analysis revealed that Mabs S abscesses in *cftr* morphant show enhanced replication of extracellular bacilli, promoting rapid bacterial expansion and tissue destruction (Figure 1F), similar to those reported in Mabs R abscesses found in WT fish (Bernut et al., 2014). Moreover, hypersusceptibility to Mabs infection in CF fish is accompanied by increased bacterial cording in Mabs R-infected animals compared with WT larvae (Figures 1G and 1H). Collectively, these results indicate that *cftr* mutants recapitulate phenotypes induced by *cftr*-MO, implying that hypersusceptibility to Mabs in *cftr* morphants is not ascribed to off-target effects but to the direct consequences of *cftr* loss, thus validating the use of *cftr*-MO to further investigate the role of CFTR in innate immunity to Mabs.

Other MABSC subsp. or the rapid-growing NTM *Mycobacterium chelonae*, which is closely related to Mabs, can be isolated from CF expectorated sputum (Harris and Kenna, 2014). Infection of *cftr* morphants with *M. abscessus* subsp. *massiliense* (Figure S2A), *M. abscessus* subsp. *bolletii* (Figure S2B), or *M. chelonae* (Figure S2C) led to increased susceptibility to infections and larval killing, similarly to Mabs-infected CFTR-defective animals (Figure 1). In contrast, neither the non-pathogenic *Mycobacterium smegmatis* (Figure S2D) nor *Mycobacterium marinum*, one of the strict pathogenic NTM and closely related to *Mycobacterium tuberculosis* (Figure S2E), induced increased larval killing in the absence of CFTR. These results indicate that the susceptibility to mycobacterial infections in CFTR-deficient embryos is specific and restricted to particular NTM species, such as those belonging to the *M. chelonae* complex (MCC), comprising MABSC and *M. chelonae*, and emphasize the protective role of CFTR in response to MCC infection by restricting bacterial pathogenesis and extracellular multiplication.

CFTR Deficiency Compromises *M. abscessus* Granuloma Maintenance by Permitting Rapid Mycobacterial Extracellular Expansion

Having previously demonstrated the importance of Mabs-induced granuloma formation and maintenance to prevent extracellular bacterial expansion and ensure the host defense (Bernut et al., 2014, 2016a), the increased mortality and extracellular mycobacterial growth in Mabs-infected CFTR-depleted animals prompted us to (1) further characterize the granulomatous response to Mabs in the absence of CFTR and (2) ask if CFTR influences the course of granuloma formation and/or granuloma composition. Mabs-infected WT, *cftr* mutants, and *cftr*

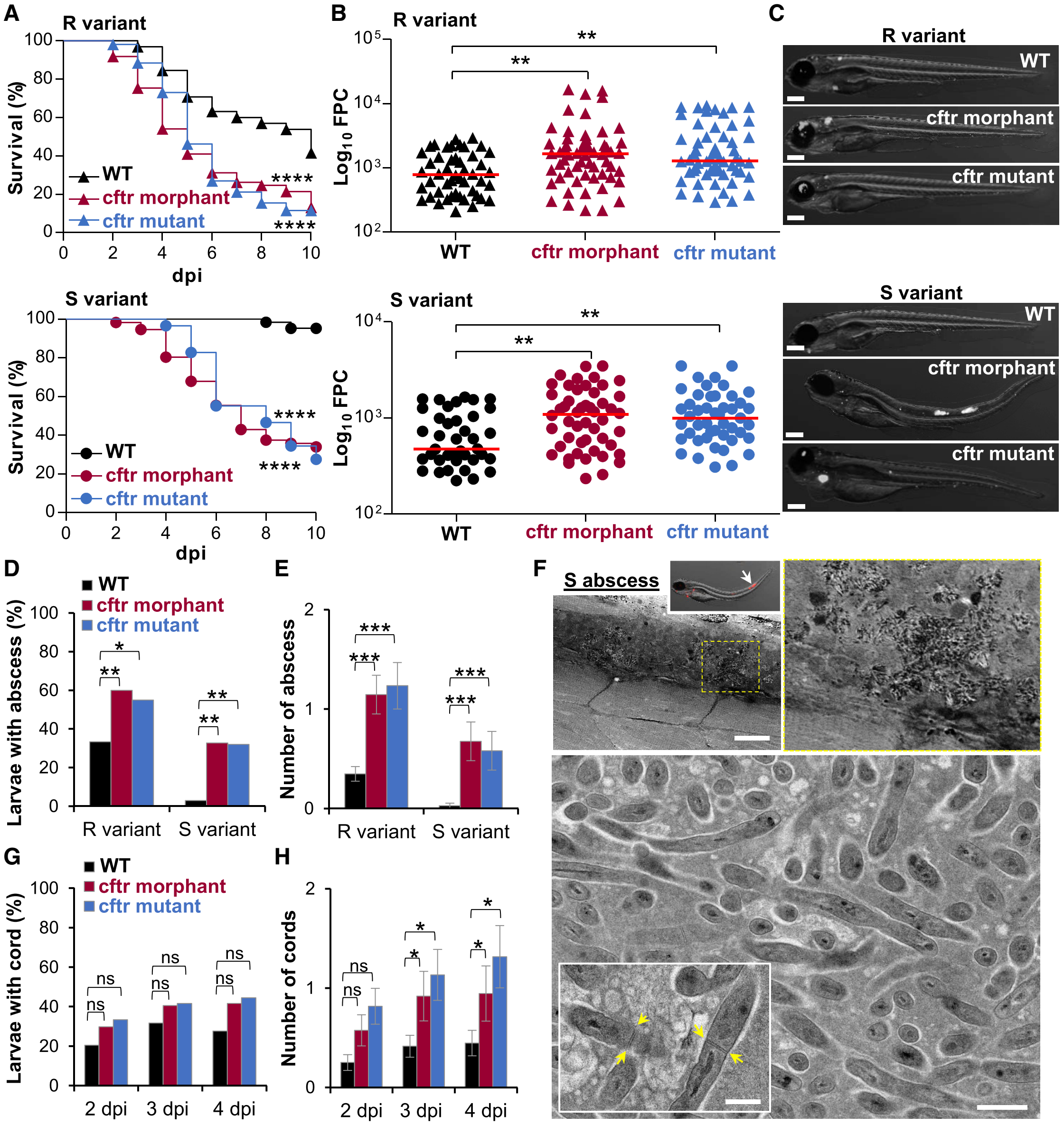

Figure 1. cftr Deficiency Increases Susceptibility to *M. abscessus* Infection

(A–H) WT, *cftr* mutant, and *cftr* morphant were intravenously (i.v.) infected with either Mabs R or S expressing tdTomato.

(A) Survival analysis of R-infected (top) or S-infected (bottom) larvae. Data are plotted as percentage of surviving animals over 10 days (n = 60, three experiments).

(B) Mean bacterial loads as fluorescence pixel counts (FPCs; average of three independent experiments) of 3 dpi larvae infected by either Mabs R (top) or S (bottom).

(C) Representative overlay fluorescence microscopy images of Mabs R-infected (top) or S-infected (bottom) larvae at 3 dpi. Scale bars, 200 μm.

(D and E) Percentage of 3 dpi larvae with abscess (D) and associated mean ± SEM number of abscess per infected animal (E) (n = 45, three experiments).

(F) EM showing a sagittal section through a Mabs S abscess in a *cftr* morphant. Overview image of the abscess into the spinal cord (top; scale bar, 25 μm). Representative EM image of the abscess lesion with many extracellular bacterial in an area of acellular necrotic debris (bottom; scale bar, 0.5 μm). Closeup showing bacterial division, with yellow arrows indicating septum of division (scale bar, 0.5 μm).

(G and H) Kinetic of Mabs R cording in whole embryos over 4 days of infection (G) and associated mean ± SEM number of cords per infected animal (H) (n = 45, three experiments).

See also Figures S1 and S2.

morphants were compared and monitored over time for granuloma formation by fluorescent microscopy. In agreement with previous observations, Mabs-granulomatous lesions were found in both the presence and absence of CFTR (Figures 2A–2G) (Tomashefski et al., 1996). However, although nascent granuloma appeared at 2 dpi and expanded in most infected WT

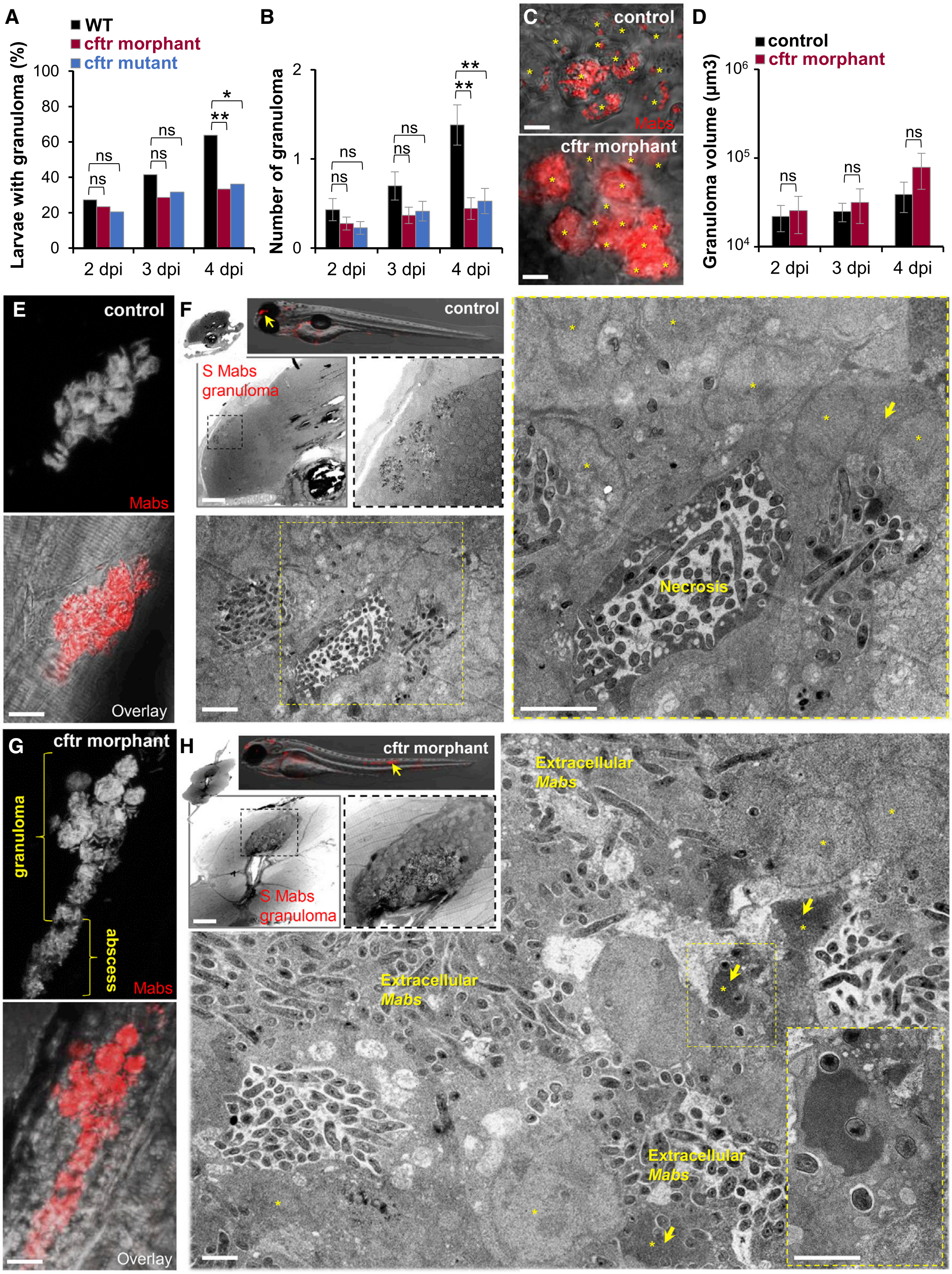

Figure 2. Absence of CFTR Impairs *M. abscessus* Infection-Mediated Granuloma Integrity and Maintenance

(A–D) WT, *cftr* mutants, and *cftr* morphants were i.v. infected with the S variant of Mabs expressing tdTomato and monitored using confocal microscopy for granuloma formation, number, and size (n = 35–45; data are plotted as mean ± SEM from three experiments).

(A and B) Kinetics of granuloma formation in whole embryos over 4 days of infection (A) and associated number of granuloma per animal (B).

(legend continued on next page)

embryos at 4 dpi (Figure 2A) (Bernut et al., 2014, 2016a), time-lapse microscopy revealed that the granuloma formation in CFTR-defective larvae remained unchanged, with fewer granulomas observed in about 20%–30% of the animals. No differences in the proportion of S or R granulomas were noticed in the context of CFTR impairment (data not shown). Additionally, confocal imaging showed that early granuloma formation and cellular aggregation events are maintained in the absence of CFTR function (Figure 2C), suggesting that granuloma elaboration proceeds regardless whether CFTR is present or not. Although this difference was not statistically significant, interestingly, at all time points, there was a trend toward increased average size of granuloma lesions in *cftr* morphants, which appeared more heavily infected than WT granulomas (Figures 2C and 2D) and continued expanding concomitantly with a time-dependent increased bacterial burden. This suggests that dysfunction of CFTR leads to a persistent acceleration in Mabs granuloma growth beyond the initial aggregation event. Next, the late granuloma-like structure in the absence of CFTR was explored by confocal imaging (Figures 2E and 2G) and EM (Figures 2F and 2H). Although the structures characterizing WT granulomas contained organized aggregates of phagocytes (Figure 2E) consisting of infected and uninfected cells surrounding a central necrotic region (Figure 2F), which efficiently control and sequester Mabs, we confirmed that impaired-CFTR granulomas are degenerated (Figures 2G and 2H), supporting the hypothesis that CFTR deficiency compromises granuloma maintenance. Overall, late CF granulomas appear poorly delimited and contain dissociated cellular aggregates (Figures 2G and 2H) with abundant extracellularly replicating bacilli forming abscesses within tissues, presumably responsible for the pronounced increase in phagocyte death observed in the granulomas (Figure 2H). In sharp contrast to WT Mabs granuloma, microscopy revealed the profusion of highly infected phagocytes in CF granulomatous lesions (Figure 2C), suggestive of impaired bacterial killing (Bernut et al., 2014, 2016a).

Together, these results indicate that a CFTR defect triggers the breakdown of Mabs granulomas typified by extracellularly growing bacteria released from dying phagocytes. This implies that CFTR is indispensable for normal granuloma structure and maintenance by controlling the phagocyte bactericidal functions and lifespan and preventing extracellular multiplication.

Dysfunction of CFTR Impairs Killing *of M. abscessus* in Macrophages

Our previous studies using Mabs-infected ZF highlighted the crucial role of macrophages for Mabs killing and infection control (Bernut et al., 2014, 2016a). In CF pathophysiology, the role of macrophages has been largely overlooked: scant evidence suggests altered macrophage properties in uncontrolled infection in CF lungs (Döring and Gulbins, 2009). To elucidate the cellular basis linking CFTR deficiency with susceptibility to Mabs infection and alteration of the macrophage functions, expression of *cftr* was knocked down in reporter lines harboring labeled macrophages. We first examined if the lack of CFTR affects chemoattraction of macrophages to the invading bacteria and/or activation of these cells, as potential mechanisms promoting extracellular bacterial growth. Leucocyte mobilization was assessed by injecting fluorescent Mabs into the hindbrain ventricle (HBV), and their phagocytic capacity was monitored after intravenous infection of the bacilli. Deficiency of *cftr* compromised neither mycobacterial-induced migration to the infected HBV (Figure 3A) nor phagocytosis (Figure 3B) at early time points, suggesting that CFTR is not required for early interactions between Mabs and macrophages. Previous studies reported that dysfunction of CFTR is associated with reduced microbicidal capacities of immune cells (Assani et al., 2017; Di et al., 2006; Duranton et al., 2012). However, recent studies have shown comparable intracellular Mabs growth in murine macrophages carrying the CFTR$^{\Delta F508}$ mutation and in WT macrophages, suggesting that functional CFTR is not required for the control of Mabs in murine macrophages infected *ex vivo* (Roux et al., 2016). Nevertheless, our microscopic observations show that CFTR ablation leads to hyperinfected phagocytes in granulomas, presumably relying on altered immune bacterial killing mechanisms (Figure 2C). Thus, to interrogate whether CFTR contributes to the macrophage mycobactericidal capacity *in vivo*, the number of Mabs in ZF macrophages was evaluated using confocal microscopy. The proportion of slightly infected (< 5 bacilli), moderately infected (5–10 bacilli), or heavily infected (> 10 bacilli) phagocytes was enumerated at 1 dpi. Compared with the control embryos, the *cftr* morphants infected by both R and S variants displayed a greater percentage of macrophages in the high-burden category (Figure 3C). This is consistent with a reduced bactericidal ability and supports the hypothesis that CFTR controls

(C) Confocal images showing a representative early Mabs granuloma in 3 dpi larvae. Scale bars, 10 μm. For a similar number of infected phagocytes (asterisk) in a granuloma, the CF granuloma contains a higher bacterial burden than the WT granuloma.

(D) Granuloma volume analysis in whole larvae over 4 days of infection.

(E–H) Control larvae or *cftr* morphants infected with Mabs S expressing tdTomato observed by confocal microscopy and EM for granuloma at 4 dpi.

(E and G) Representative confocal imaging of a granuloma within the spinal cord of an infected animal showing the development of a compact and organized phagocyte aggregate moderately infected in control fish (E) compared with a *cftr* morphant harboring a heavily loaded granuloma whose disruption leads to bacterial spread characterized by abscess formation (G). Scale bars, 10 μm.

(F) EM showing a sagittal section through a WT Mabs granuloma. Overview image of granuloma (arrow) into the brain of infected WT (top; scale bar, 50 μm). Representative EM image of a compact and well-organized WT granuloma (bottom and right; processed as stitching of micrographs; scale bars, 2 μm) showing a stable mycobacterial-containing structure with a central necrotic area and a typical dense region of cellular debris and surrounded by numerous infected phagocytes (asterisk) and giant cells (arrow).

(H) EM showing a sagittal section through a Mabs granuloma-like lesion in absence of CFTR. Overview image of granuloma in the spinal cord (arrow) of infected *cftr* morphant (top; scale bar, 25 μm). Representative EM image of a CFTR-depleted granuloma (bottom and right; processed as stitching of micrographs; scale bar, 2 μm) showing a necrotic structure with replicating bacteria and numerous dead infected phagocytes harboring a typical apoptotic nucleus (asterisk) with chromatin “superaggregation.” Most bacilli stay extracellular.

OPEN
ACCESS
CellPress

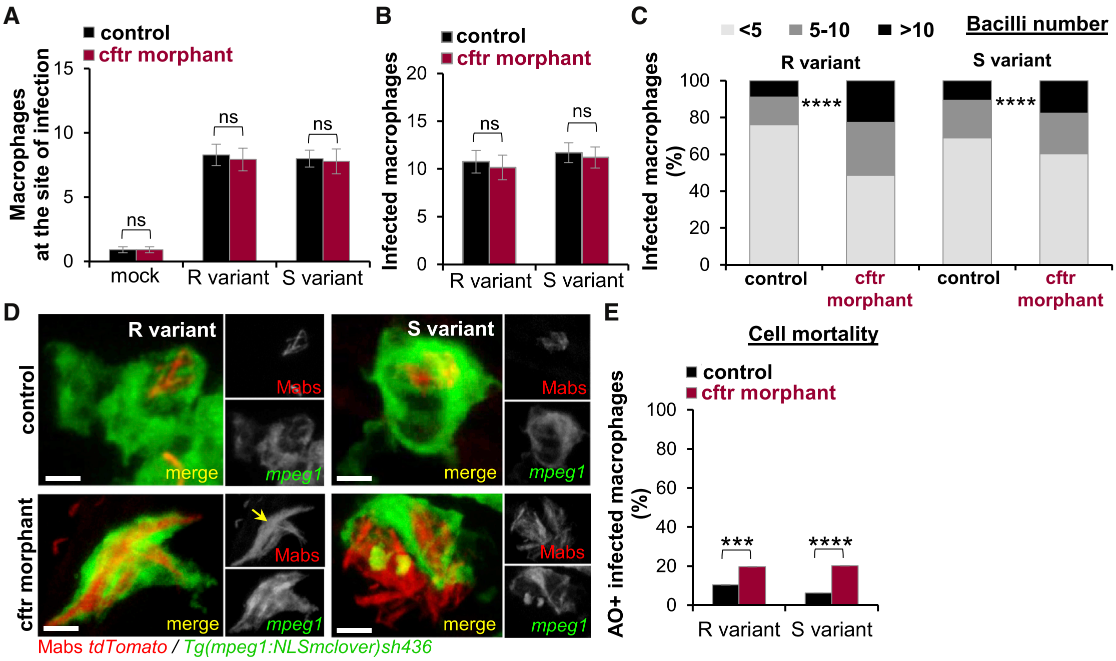

Figure 3. *cftr* Knockdown Diminishes Intracellular Killing of *M. abscessus* and Promotes Macrophage Death

(A) Control and *cftr* morphants Tg(mpeg1:NLSmclover)sh436 were infected with Mabs R or S expressing tdTomato into the hindbrain ventricle (HBV). Confocal microscopy was used to monitor the cell recruitment at 2 hpi. Mean ± SEM number of macrophages recruited to the infected HBV (n = 20, two experiments).

(B) mpeg1:NLSmclover control and *cftr* morphants were i.v. infected with Mabs R or S expressing tdTomato. Mean ± SEM number of infected macrophages in the caudal hematopoietic tissue (CHT) at 4 hpi (n = 20, two experiments).

(C and D) mpeg1:NLSmclover control and *cftr* morphants were i.v. infected with Mabs R or S expressing tdTomato imaged at 1 dpi using confocal microscopy to quantify the intracellular bacterial loads.

(C) Average proportions of infected macrophages containing fewer than five, five to ten, or more than ten bacteria in the CHT (n = 16, two experiments).

(D) Confocal images showing infected macrophages. Although WT-macrophages efficiently contain intracellular bacilli, CF macrophages fail to control Mabs growth. Arrow indicates intracellular Mabs R cording. Scale bars, 2 μm.

(E) Control and *cftr* morphants Tg(mpeg1:mCherry-F)ump2 were i.v. infected with Mabs R or S expressing E2-Crimson and stained with acridine orange (AO). Dead infected macrophages in the CHT were counted using confocal microscopy at 2 dpi. Data are plotted as mean ± SEM from two experiments (n = 20–22).

intracellular growth and killing of Mabs. Because death of Mabs-infected phagocytes releases and propagates free bacilli in the extracellular milieu (Bernut et al., 2014), we examined the extent of macrophage death in infected larvae. Combined confocal observations and quantification of acridine orange (AO)-positive infected macrophages shows that infection in *cftr* morphants is characterized by the presence of heavily infected phagocytes with an impaired ability to restrict bacterial growth, occurring prior to cell death (Figure 3D). Although the basal levels of dead macrophages were equal between the PBS-injected control embryos and *cftr* morphants (data not shown), enumeration of AO-labeled macrophages infected with either R or S confirms higher yields of dead phagocytes in *cftr* morphants at 2 dpi compared with the control embryos at 2 dpi (Figure 3E). This agrees with the presence of apoptotic immune cells seen in CF granulomatous lesions (Figure 2). Importantly, the proportion of dead macrophages was lower in WT embryos infected with S- compared with R-infected embryos (Bernut et al., 2014; Roux et al., 2016) but remained equal in the absence of CFTR (Figure 3E), substantiating the crucial role of CFTR in containing intracellular *Mabs*.

Modulation of the Neutrophilic Response to *M. abscessus* by CFTR

The chronic infections with a neutrophilic inflammation are a hallmark of CF lung pathophysiology (Cantin, 1995). Having reported that Mabs lesions are characterized by an influx of neutrophils and that these cells are critical in the host defense against Mabs infections (Bernut et al., 2014, 2016a; Malcolm et al., 2018), we next addressed whether CFTR ablation influences the behavior of neutrophils by examining and comparing the dynamic of leucocyte mobilization in WT and CFTR-deficient larvae using the *Tg(mpx:GFP)i114* transgenic line labeling neutrophils with GFP (Renshaw et al., 2006). Surprisingly, CFTR deficiency strongly reduced neutrophil mobilization toward the infection sites, upon local injection of either R or S variants, as revealed by microscopy observations and quantitative analysis of the number of neutrophils at the site of infection at 4 h post-infection (hpi) in *cftr* morphant (Figures 4A–4D) and despite a larger baseline number of neutrophils in the absence of CFTR (Figures S3A and S3B). These phenotypes are in line with the impaired neutrophil trafficking in *cftr* morphants infected with *P. aeruginosa* (Phennicie et al., 2010).

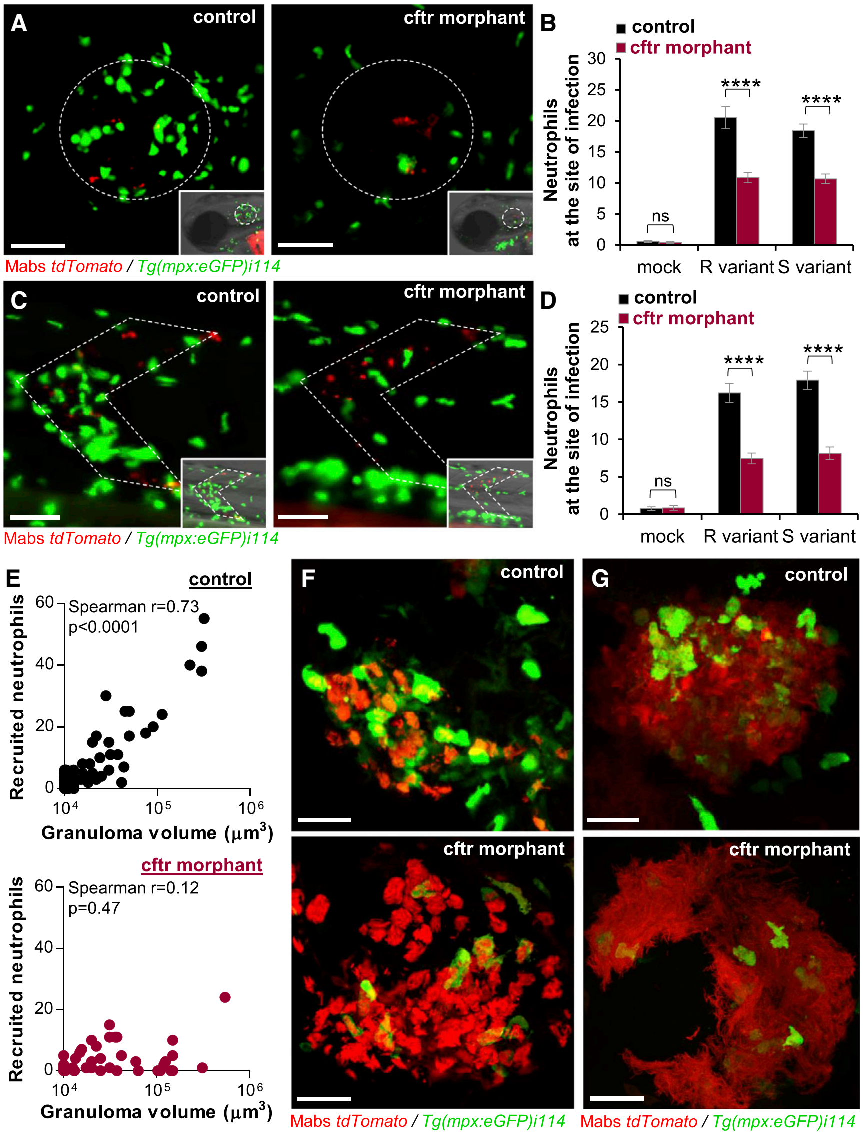

Figure 4. CFTR Deficiency Reduces Chemoattraction of Neutrophils to Infection Sites

(A–G) Controls and *cftr* morphants *Tg(mpx:GFP)i114* were infected in the otic cavity (A and B), the muscle (C and D), or the caudal vein (E–G) with Mabs R or S expressing tdTomato and monitored using confocal microscopy to follow the neutrophil behavior toward infection sites.

(A–D) Representative images (A and C) and mean ± SEM number (B and D) of neutrophils recruited to infection sites after 3 hpi (n = 30, three experiments). Scale bars, 50 μm.

(E) Number of neutrophils recruited to WT (top) or CFTR-depleted (bottom) nascent granulomas as a function of granuloma volume.

(F and G) Confocal images showing the distribution of a neutrophil-associated granuloma (F) and abscess (G) in a control animal versus a *cftr* morphant. Scale bars, 25 μm.

See also Figures S3–S5.

Additionally, confocal microscopy unraveled impaired neutrophil mobilization around and into Mabs-granulomatous lesions (Figures 4E and 4F) and abscesses (Figure 4G) in the *cftr* morphants. We previously established the linear relationship between the number of recruited neutrophils and the Mabs granuloma volume in WT ZF (Bernut et al., 2016a). Although WT granulomas contained numerous neutrophils distributed into and on the periphery of these cellular structures, CF granulomas were dominated mostly by macrophages and contained fewer neutrophils (Figures 4E and 4F).

CF neutrophils remain capable of engulfing bacteria similarly to WT neutrophils (Figure S4A), suggesting that their phagocytosis activity toward Mabs is independent of CFTR. However, they fail to control intracellular Mabs (Figure S4B), leading to increased cell death (Figure S4C), as reported for macrophages (Figure 3). To determine whether the impaired neutrophilic response to Mabs infection in *cftr*-deficient ZF is linked to a possible intrinsic alteration in neutrophil recruitment, we performed a neutrophil mobilization assay using fMLP, a synthetic neutrophil chemoattractant. Injection of fMLP into the otic vesicle induced similar neutrophil recruitment in *cftr* morphants and control larvae (Figure S5A). Previous findings highlighted also the requirement of IL-8 for neutrophil trafficking during Mabs infections and its crucial role in elaborating Mabs granuloma (Bernut et al., 2016a). That CFTR impairs the early mobilization of neutrophils into Mabs lesion led us to enquire whether CFTR depletion influences *cxcl8* expression. qRT-PCR analysis revealed a similar level of *cxcl8* expression in *cftr* morphants and control animals infected with both S and R variants (Figure S5B), indicating that impaired neutrophil trafficking is not caused by an alteration in the Cxcl8 pathway-mediated neutrophil mobilization.

Overall, these results suggest CFTR is required for early and late neutrophil recruitment to localized Mabs infection and to control the intracellular growth of Mabs.

Intracellular Killing of *M. abscessus* Is Mediated by NOX2-Dependent ROS Production

Mabs induces an oxidative stress response with the generation of intracellular reactive oxygen species (ROS) by macrophages and neutrophils (Bernut et al., 2016a; Malcolm et al., 2018). Loss of functional CFTR reduces the macrophage respiratory burst response and impairs killing of intracellular *B. cenocepacia* (Assani et al., 2017). Thus, intracellular ROS generation in CFTR-deleted larvae was investigated as a plausible mechanism through which CFTR-mediated oxidative stress controls Mabs infections. Because production of intracellular ROS by professional

OPEN
ACCESS
CellPress

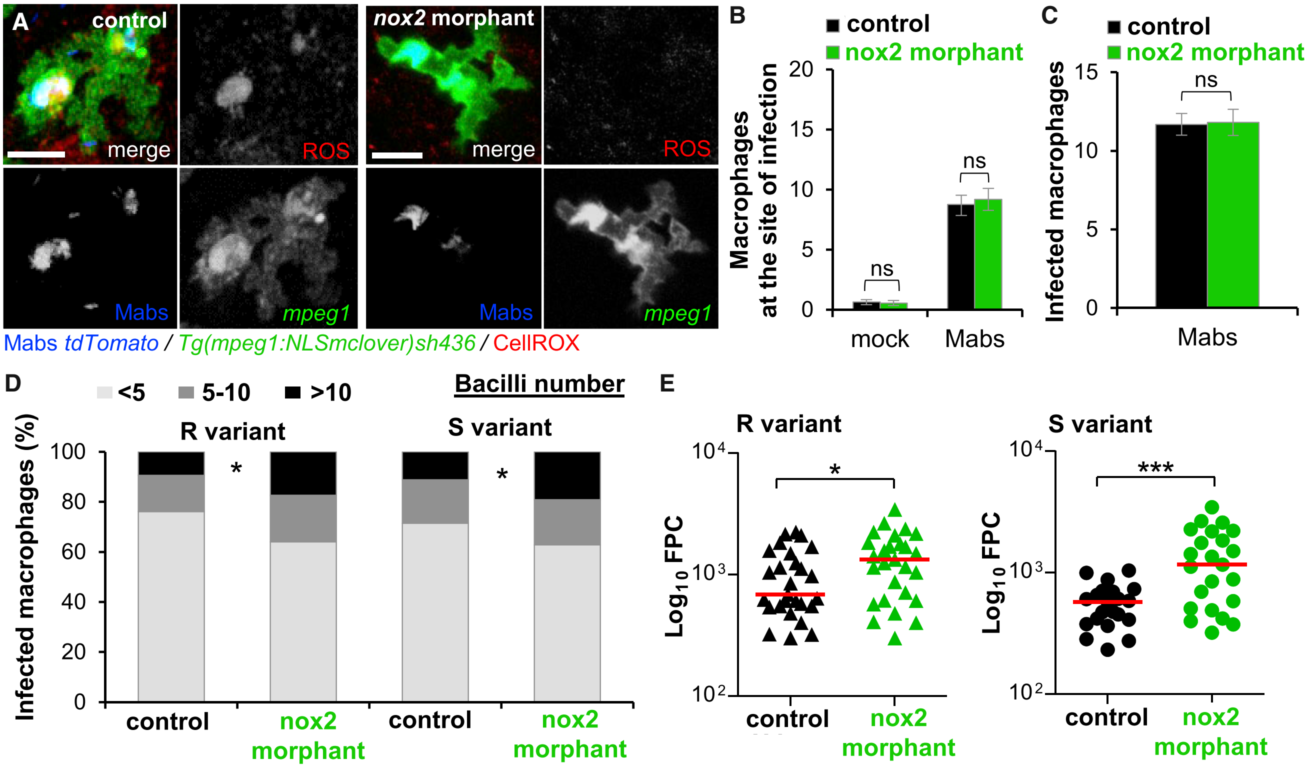

Figure 5. NADPH Oxidase-Mediated Intracellular ROS Production Restricts *M. abscessus* Growth

(A) mpeg1:NLSmclover controls and *nox2* morphants were infected with Mabs expressing tdTomato and stained for ROS production using CellROX deep red. Representative ROS-producing infected macrophages revealed by confocal microscopy in a *nox2* morphant versus a WT larvae. Scale bars, 5 μm.

(B) mpeg1:NLSmclover controls and *nox2* morphants were infected with Mabs expressing tdTomato into the HBV and monitored using confocal microscopy to analyze cell recruitment. Mean ± SEM number of macrophages recruited to the infected HBV at 2 hpi (n = 20, two experiments).

(C–E) mpeg1:NLSmclover controls or *nox2* morphants were i.v. infected with Mabs S (C and D) or R (D) expressing tdTomato.

(C) Mean number of infected macrophages in the CHT at 4 hpi (n = 20, two experiments).

(D) Average proportions of infected macrophages containing fewer than five, five to ten, or more than ten bacteria in the CHT at 1 dpi (n = 16, two experiments).

(E) Mean FPC of 3 dpi larvae i.v. infected by either Mabs R (left) or S (right) expressing tdTomato from three experiments.

phagocytes relies mainly on *nox2* (Warnatsch et al., 2017), knockdown experiments abolishing initial NOX2-mediated ROS production (Figure 5A) were conducted using a specific MO targeting *nox2* (Roca and Ramakrishnan, 2013). Although injection of *nox2*-MO failed to affect early macrophage mobilization into the HBV (Figure 5B) or macrophage phagocytosis (Figure 5C), it did lead to larger numbers of heavily infected cells (Figure 5D). Consistent with these findings, global mycobacterial loads increased in the absence of NOX2 (Figure 5E). Consistently, the *nox2* morphants developed more abscesses than the control embryos, leading to the premature larval death (data not shown). This implies that reduced ROS production in *nox2* morphants is deleterious for the host and that the enhanced susceptibility of these fish to the infection highlights the key role of the NADPH-mediated intracellular ROS for clearing Mabs.

To determine whether an impaired oxidative defense is involved in defective CFTR-associated reduced bacterial killing in early infection stages, we examined whether heat-killed and intact Mabs induce intracellular ROS production in macrophages using the CellROX dye. ROS-labeled phagocytes were rapidly detected, but the number of ROS-positive macrophages harboring either living or heat-killed R or S variants was lower in *cftr* morphant than in control ZF at 2 hpi (Figures 6A and 6B), consistent with the altered ROS production reported in the CF context (Assani et al., 2017; Phennicie et al., 2010). Of note, the larger number of ROS-positive macrophages infected with heat-killed bacteria suggests that Mabs has developed mechanisms to overcome the host oxidative killing mechanisms. Moreover, similar results were observed regarding reduced ROS production in CF-infected neutrophils (Figures S6A and S6B). To further inquire whether the impaired intracellular ROS production in absence of CFTR is linked to a possible decrease in NADPH oxidase activity, the NOX2-mediated ROS signaling pathway was dissected in defective *cftr* embryos. Mabs infection triggers an upregulation of *nox2* in control fish and qRT-PCR analysis confirmed a reduced *nox2* expression in *cftr* morphants (Figure 6C), suggesting that CFTR orchestrates the early regulation of ROS induction by modulating the NOX2/NADPH oxidase activity. That the intracellular bacterial profiles were similar in the *cftr* morphants and in the double *cftr/nox2* morphants (Figure 6D) suggests that the enhanced intracellular bacterial growth in *cftr* morphants is directly linked to defective NOX2-NADPH

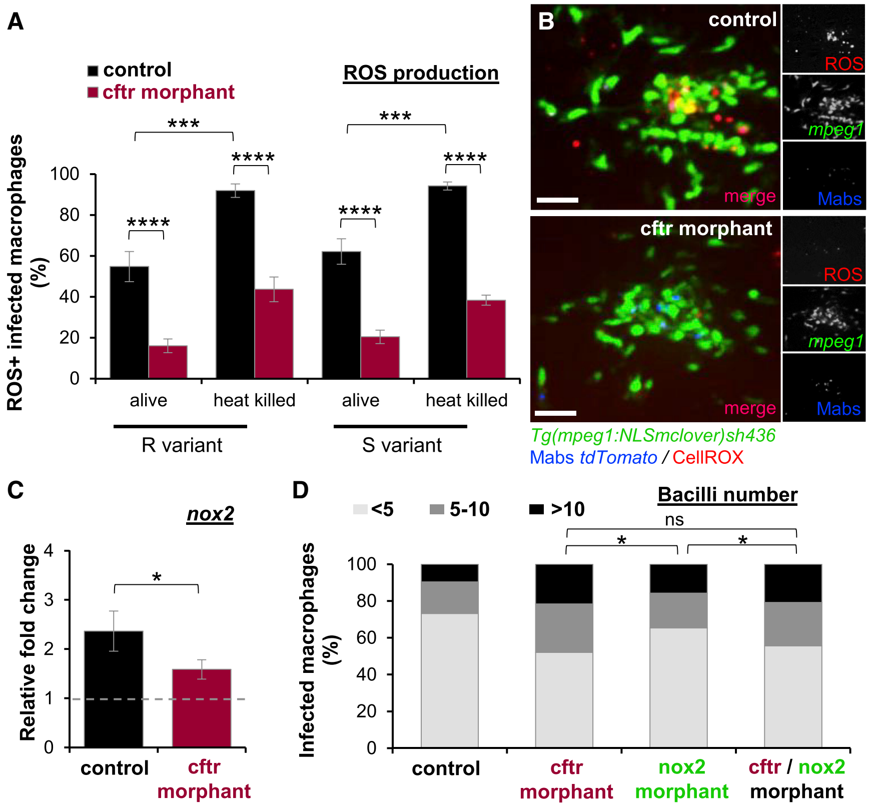

Figure 6. CFTR Modulates NADPH Oxidase-Mediated ROS Production

(A and B) mpeg1:NLSmclover controls or *cftr* morphants were infected with either living or heat-killed Mabs R or S expressing tdTomato into the muscle and stained for ROS production using CellROX deep red and analyzed using confocal microscopy.

(A) Proportion of ROS-positive infected macrophages at 2 hpi (n = 16, two experiments).

(B) Distribution of representative ROS-producing macrophages within the muscle at 2 hpi. Scale bars, 15 μm.

(C) qRT-PCR measurement in whole embryos i.v. infected with Mabs and plotted as fold increase over mock injection for *nox2*. Mean relative ± SEM gene expression of three independent replicates.

(D) mpeg1:NLSmclover controls, *cftr*, *nox2*, and double *cftr/nox2* morphants were i.v. infected with Mabs expressing tdTomato, and intracellular bacterial loads were quantified at 1 dpi using confocal microscopy. Graph represents the average proportions of infected macrophages containing fewer than five, five to ten, or more than ten bacteria in the CHT (n = 15, two experiments).

See also Figures S6 and S7.

oxidase-related ROS production. Overall, these results demonstrate that CFTR deficiency alters NOX2/NADPH oxidase-dependent ROS production that, in turn, fails to restrict intracellular growth of Mabs and that the release of NOX2-derived ROS participates in clearing Mabs in both macrophages and neutrophils (Figures 6 and S6).

Activated macrophages restrict mycobacterial growth through a TNF-mediated ROS-dependent pathway (Bernut et al., 2016a; Dewas et al., 2003). Combined with the fact that CFTR dysfunction is associated with alterations in the innate immune regulation network and pro-inflammatory cascades (Döring and Gulbins, 2009; Cantin, 1995), we next explored whether abnormal *tnf* induction could be involved in reduced ROS-mediated bacterial killing in *cftr* morphants during the early stages of infection. To assess the effect of *cftr* loss of function on *tnf*α production by Mabs-infected macrophages, Mabs E2-Crimson were injected into the muscle of either controls or *cftr* morphants *tnfa:GFP-F/mpeg1:mCherry-F* double transgenic. Microscopy observations indicate that both WT or CF animals exhibited equal proportions of GFP-positive infected phagocytes containing either Mabs S or R at 4 hpi (Figures S7A and S7B), suggesting that the early impaired ROS generation associated with CFTR ablation is TNF independent. At later stages, however, CFTR ablation triggers a hyper-inflammatory response following infection with Mabs, with qRT-PCR revealing upregulation of *tnf*α expression, especially after infection with the R form (Figure S7C), similar to findings reported previously in mice (Catherinot et al., 2009).

Collectively, these data indicate that NOX2/NADPH oxidase-dependent ROS production by infected phagocytes represents a critical host defense mechanism against Mabs and suggest that the inherent deficit in NOX2-derived oxidative stress in CF leukocytes is responsible for their defective bacterial killing responses.

DISCUSSION

Pulmonary disease is the leading cause of morbidity and mortality in CF and is characterized by a vicious circle of chronic infections and persistent inflammation. Among the deleterious bacteria found in CF airway, the fast growing multidrug-resistant Mabs has emerged as an important respiratory pathogen of major concern in CF centers worldwide (Parkins and Floto, 2015). However, our understanding of the particular vulnerability of CF patients to Mabs infection remains limited by the lack of suitable animal models mimicking the immune abnormalities found in the CF population. Nevertheless, important insights into the pathophysiology of Mabs diseases have recently been obtained in the ZF (Bernut et al., 2014, 2016a), and the very close structural relatedness between ZF and human CFTR emphasizes further the relevance of ZF to study CFTR functions (Zhang and Chen, 2016; Liu et al., 2017). To address these unmet needs, we exploited here CF ZF as an innovative vertebrate recapitulating aspects of CF immuno-pathogenesis. Thanks to genetic and high-resolution imaging approaches, we report the direct stepwise dissection of Mabs infection in an animal lacking CFTR to elucidate the biological implication of CFTR in innate immunity to Mabs.

Mabs-infected CFTR-depleted ZF rapidly succumb to infection, reflecting a hypersusceptibility to this mycobacterium in CF, providing a first glimpse into CFTR-mediated host defenses

OPEN
ACCESS
CellPress

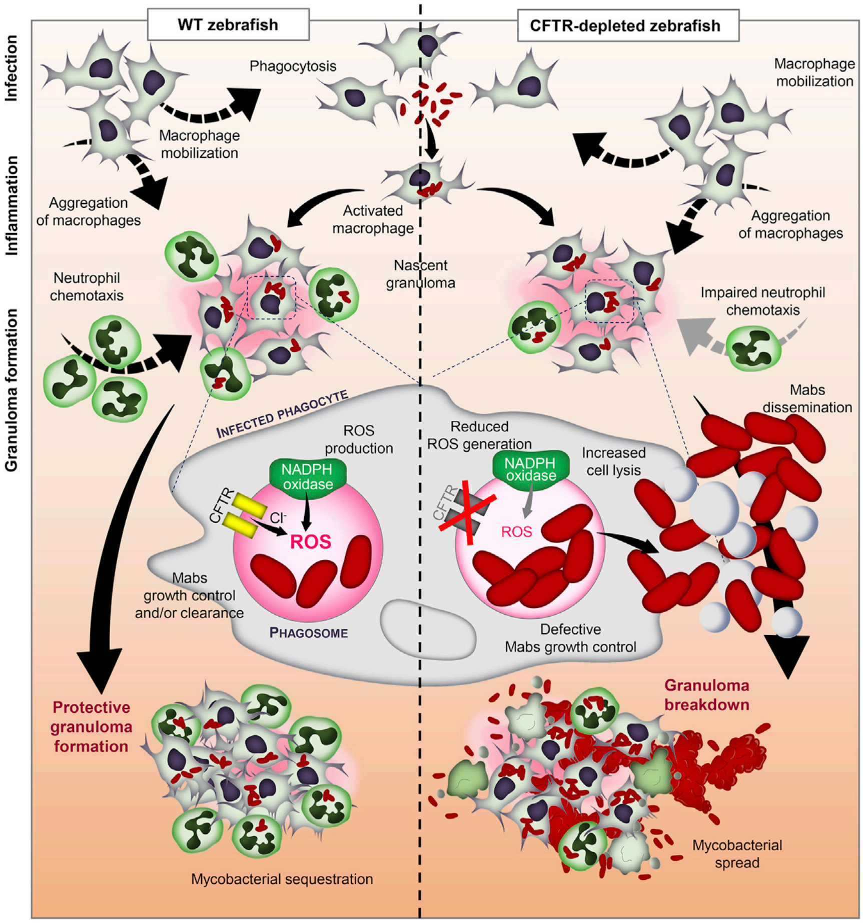

Figure 7. Critical Role of the CFTR/NADPH Oxidase Axis for Efficient ROS Production and Protective Immunity against *M. abscessus* Infection

Schematic overview summarizing the CFTR/NOX2 axis-dependent ROS production in Mabs infection control.

to Mabs infection. Mechanisms leading to the formation of protective Mabs granuloma depend on efficient macrophage and neutrophil cooperation, orchestrated by fine-tuning innate immune responses (Bernut et al., 2016a). The spatiotemporal events associated with CFTR ablation (Figure 7) reveal a mechanism whereby CFTR participates in neutrophil chemotaxis to the infected sites and the adjustment of oxidative host defenses, conditioning efficient phagocyte-mediated bacterial killing, together generating a protective granulomatous response. Infection of ZF with Mabs is characterized by (1) rapid engulfment of the bacilli by macrophages; (2) activation of macrophages, resulting in chemotaxis guiding neutrophils to pre-forming granulomas and ROS production by NOX2 for intracellular killing of Mabs; and (3) homeostatic granuloma formation to sequester Mabs, containing the infection and favoring the development of chronic disease. Conversely, CFTR deletion promotes increased susceptibility to Mabs infections, correlating with (1) deficiency in ROS production altering phagocyte-mediated Mabs killing, resulting in increased intracellular bacterial loads and premature cell death, and (2) impaired neutrophil chemotaxis toward nascent granulomas. These two factors conspire to alter the maintenance of protective granuloma with uncontrolled extracellular mycobacterial spread, conducting to acute infection and larval death. Other studies suggested that dysfunction of CFTR dampens the microbicidal activity of immune cells (Assani et al., 2017; Di et al., 2006; Duranton et al., 2012), promoting infectious pathology in CF airways. However, existing CF models have failed to reproduce the hypersusceptibility phenotype associated with mycobacterial infections in CF (Roux et al., 2016; Le Moigne et al., 2015). Using the CF ZF model, we report here that primary alterations in innate immunity directly contribute to increased susceptibility to the infection. Whereas ROS produced by the NADPH oxidase during the respiratory burst participate in the elimination of pathogens, Mabs has been reported to withstand the hostile oxidative environments inside phagocytes (Oberley-Deegan et al., 2010), although this awaits *in vivo* confirmation. In ZF, inhibition of the NOX2/NADPH oxidase pathway enhances intracellular growth. Given the importance of NOX2-derived ROS production in Mabs killing, a reduced oxidative response in CF ZF is very likely to explain the increase susceptibility to Mabs. Of note, the bacterial burden in *nox2*-defective macrophages is lower than in CFTR macrophages (Figure 6D), suggesting that additional CFTR-mediated mechanisms are likely to participate in Mabs clearance.

Overly exuberant neutrophil influx associated with harmful oxidative stress is a hallmark of the inflammatory CF lungs (Cantin, 1995; Hector et al., 2014). The increased number of neutrophils in *cftr* morphants mirrors the neutrophilia seen in CF. Our results emphasize also the neutrophil chemotaxis impairment to Mabs, as shown previously in *P. aeruginosa*-infected ZF (Phennicie et al., 2010) but raising also controversial questions regarding inflammatory and infectious CF pathologies. Supporting the view that neutrophilic inflammation-mediated Mabs infection plays a critical role in host defense against this pathogen by maintaining granuloma integrity and preventing extracellular bacterial multiplication (Bernut et al., 2016a), we show that the capacity of neutrophils to migrate in a CFTR-dependent manner is involved in the formation of protective granulomas. Release of ROS by epithelial cells through NOX2-NADPH oxidase has been implicated in neutrophil chemotaxis to wounds (Braunersreuther et al., 2013). We provide here evidence for CFTR in modulating the NOX2 oxidative pathway, in which a

local redox imbalance in *cftr* morphants may account for the reduced number of infiltrated neutrophils toward Mabs. This suggests that NOX2-dependent ROS production from activated leucocytes triggers an oxidative environment sustaining neutrophilic mobilization to efficiently contain bacteria within homeostatic granulomas. The NOX/DUOX family NADPH oxidase-mediated oxidative defenses play a critical role to control invading pathogens by triggering neutrophil chemotaxis to infected tissues or bacterial killing (Brothers et al., 2013; Rada and Leto, 2008). In addition to the reduced *nox2* expression in absence of CFTR, presumably contributing to imbalanced adjustments of redox signaling, other mechanisms altering ROS production via other NADPH oxidase complexes by epithelial or immune cells may also contribute to the impaired bactericidal function and/or chemotaxis of leucocytes during Mabs infection.

The clinical relevance of ROS production in host defense, notably the NOX2/NADPH oxidase in granuloma formation, is consistent with mutations in *nox2* leading to chronic granulomatous disease (CGD) typified by the development of large size and poorly structured granulomas that are unable to sequester mycobacteria (Deffert et al., 2014) and associated with severe inflammation (Rieber et al., 2012). However, although the altered NADPH oxidase function in CGD can lead to increased disease severity following infection with *M. tuberculosis* or vaccination with *M. bovis* BCG (Deffert et al., 2014), reports of infection with NTM remain anecdotal (Ohga et al., 1997; Weening et al., 2000; Chusid et al., 1975), and no particular link with *M. abscessus* has been ascribed yet.

This, together with clinical cases of CF patients heavily infected with Mabs R (Jönsson et al., 2007; Catherinot et al., 2009), demonstrates that Mabs exacerbates inflammation in absence of CFTR, implicating a critical inflammatory pathology associated with tissue damage and persistent Mabs infections. Thus, at the later stages of infection, it is possible that an imbalance of ROS production or neutrophil chemotaxis could be caused by CF-mediated hyperinflammation acting as a negative feedback loop that would undo the fine-tuning of immune responses.

Our study indicates that *cftr* is a regulator of host immunity to MABSC but not to other saprophytic (*M. smegmatis*) or pathogenic (*M. marinum*) NTM, suggesting that species-specific restriction mechanisms may exist for these organisms. In fact, we show that *M. marinum*-infected *cftr* morphants succumbed to infection more slowly than controls, suggesting that inactivation of *cftr* triggers a mild protective immunity against tuberculosis. Indeed, it has been proposed that the high carrier rate for CFTR mutations among Caucasians is due to an evolutionary selective advantage against infectious disease, with candidate agents including cholera (Gabriel et al., 1994), typhoid fever (Pier et al., 1998), and tuberculosis (Meindl, 1987), in which the pandemic in the early 1600s could explain the modern-day CF incidence rates in European-descendent populations (Poolman and Galvani, 2007). Recent studies support the hypothesis that carrying the most common F508del *cftr* allele protects against *M. tuberculosis* infection (Bosch et al., 2017). This species-specific susceptibility to different mycobacteria is particularly intriguing and deserves further attention.

In summary, we demonstrate that CFTR dysfunction leads to hypersusceptibility to *Mabs* infection *in vivo*, potentially explaining the high rates of infection seen clinically in CF patients. We anticipate that insights obtained from ZF may guide the development of future therapies targeting innate immune defects in CF.

STAR★METHODS

Detailed methods are provided in the online version of this paper and include the following:

- KEY RESOURCES TABLE
- CONTACT FOR REAGENT AND RESOURCE SHARING
- EXPERIMENTAL MODELS AND SUBJECT DETAILS
  - Bacterial Strains
  - Zebrafish Husbandry and Ethic statements
- METHOD DETAILS
  - Creation of theTg(mpeg1:NLSmClover) transgenic line
  - Morpholino injection
  - Zebrafish infection
  - qRT-PCR
  - Epifluorescence, Confocal Microscopy and Imaging
  - Transmission Electron Microscopy
  - Neutrophils Recruitment Assay
  - Cell Death and Reactive Oxygen Species Detection
- QUANTIFICATION AND STATISTICAL ANALYSIS

SUPPLEMENTAL INFORMATION

Supplemental Information includes one table and seven figures and can be found with this article online at https://doi.org/10.1016/j.celrep.2019.01.071.

ACKNOWLEDGMENTS

This study was supported by the European Community's Horizon 2020—Research and Innovation Framework Programme (H2020-MSCA-IF-2016) under the Marie-Curie IF CFZEBRA (751977) to A.B., the Biotechnology and Biological Sciences Research Council (BBSRC), United Kingdom, under a project grant (BB/L000830/1) to N.V.O., a Medical Research Council (MRC), United Kingdom, Programme Grant (MR/M004864/1) to S.A.R., the French National Research Agency (DIMYVIR ANR-13-BSV3-0007-01) to J.-L.H. and L.K., and Fondation pour la Recherche Médicale (FRM) (DEQ20150331719), France, to L.K. We would like to thank Michel Bagnat for kindly providing us with *cftr*^{pd1049} mutant and *gBAC(cftr-RFP)pd1042* line and Georges Lutfalla for designing the *cftr*-MO. We thank the ZF facilities at Bateson Centre and at CNRS-CRBM. We wish to thank the CEMIPAI, the Wolfson Light Microscopy Facility (MRC grant G0700091), and Montpellier RIO Imaging facilities. We also thank the University of Sheffield and the University of Montpellier for support.

AUTHOR CONTRIBUTIONS

A.B. and L.K. conceived the study and wrote the manuscript with input from J.-L.H., R.A.F., and S.A.R., A.B., S.A.R., and L.K. designed experiments and analyzed data. A.B. performed experiments and produced the figures. C.D. performed PCR experiments. N.V.O. made and provided the *Tg(mpeg1:NLSclover)*sh436 line. A.N. performed EM preparation and imaging experiments. All authors reviewed the manuscript.

DECLARATION OF INTEREST

The authors declare no competing interests.

Received: October 12, 2018
Revised: December 17, 2018
Accepted: January 17, 2019
Published: February 12, 2019

SUPPORTING CITATIONS

The following reference appears in the Supplemental Information: Weaver et al. (2016).

REFERENCES

Assani, K., Shrestha, C.L., Robledo-Avila, F., Rajaram, M.V., Partida-Sanchez, S., Schlesinger, L.S., and Kopp, B.T. (2017). Human cystic fibrosis macrophages have defective calcium-dependent protein kinase C activation of the NADPH oxidase, an effect augmented by *Burkholderia cenocepacia*. J. Immunol. *198*, 1985–1994.

Bernut, A., Herrmann, J.L., Kissa, K., Dubremetz, J.F., Gaillard, J.L., Lutfalla, G., and Kremer, L. (2014). *Mycobacterium abscessus* cording prevents phagocytosis and promotes abscess formation. Proc. Natl. Acad. Sci. U S A *111*, E943–E952.

Bernut, A., Dupont, C., Sahuquet, A., Herrmann, J.L., Lutfalla, G., and Kremer, L. (2015). Deciphering and imaging pathogenesis and cording of *Mycobacterium abscessus* in zebrafish embryos. J. Vis. Exp. *9*, 103.

Bernut, A., Nguyen-Chi, M., Halloum, I., Herrmann, J.L., Lutfalla, G., and Kremer, L. (2016a). *Mycobacterium abscessus*-induced granuloma formation is strictly dependent on TNF signaling and neutrophil trafficking. PLoS Pathog. *12*, e1005986.

Bernut, A., Viljoen, A., Dupont, C., Sapriel, G., Blaise, M., Bouchier, C., Brosch, R., de Chastellier, C., Herrmann, J.L., and Kremer, L. (2016b). Insights into the smooth-to-rough transitioning in *Mycobacterium bolletii* unravels a functional Tyr residue conserved in all mycobacterial MmpL family members. Mol. Microbiol. *99*, 866–883.

Bosch, L., Bosch, B., De Boeck, K., Nawrot, T., Meyts, I., Vanneste, D., Le Bourlegat, C.A., Croda, J., and da Silva Filho, L.V.R.F. (2017). Cystic fibrosis carriership and tuberculosis: hints toward an evolutionary selective advantage based on data from the Brazilian territory. BMC Infect. Dis. *17*, 340.

Braunersreuther, V., Montecucco, F., Asrih, M., Pelli, G., Galan, K., Frias, M., Burger, F., Quinderé, A.L., Montessuit, C., Krause, K.H., et al. (2013). Role of NADPH oxidase isoforms NOX1, NOX2 and NOX4 in myocardial ischemia/reperfusion injury. J. Mol. Cell. Cardiol. *64*, 99–107.

Brothers, K.M., Gratacap, R.L., Barker, S.E., Newman, Z.R., Norum, A., and Wheeler, R.T. (2013). NADPH oxidase-driven phagocyte recruitment controls *Candida albicans* filamentous growth and prevents mortality. PLoS Pathog. *9*, e1003634.

Bryant, J.M., Grogono, D.M., Greaves, D., Foweraker, J., Roddick, I., Inns, T., Reacher, M., Haworth, C.S., Curran, M.D., Harris, S.R., et al. (2013). Whole-genome sequencing to identify transmission of *Mycobacterium abscessus* between patients with cystic fibrosis: a retrospective cohort study. Lancet *381*, 1551–1560.

Cantin, A. (1995). Cystic fibrosis lung inflammation: early, sustained, and severe. Am. J. Respir. Crit. Care Med. *151*, 939–941.

Catherinot, E., Roux, A.L., Macheras, E., Hubert, D., Matmar, M., Dannhoffer, L., Chinet, T., Morand, P., Poyart, C., Heym, B., et al. (2009). Acute respiratory failure involving an R variant of *Mycobacterium abscessus*. J. Clin. Microbiol. *47*, 271–274.

Chusid, M.J., Parrillo, J.E., and Fauci, A.S. (1975). Chronic granulomatous disease. Diagnosis in a 27-year-old man with *Mycobacterium fortuitum*. JAMA *233*, 1295–1296.

Clatworthy, A.E., Lee, J.S., Leibman, M., Kostun, Z., Davidson, A.J., and Hung, D.T. (2009). *Pseudomonas aeruginosa* infection of zebrafish involves both host and pathogen determinants. Infect. Immun. *77*, 1293–1303.

DaCosta, A., Jordan, C.L., Giddings, O., Lin, F.C., Gilligan, P., and Esther, C.R., Jr. (2017). Outcomes associated with antibiotic regimens for treatment of *Mycobacterium abscessus* in cystic fibrosis patients. J. Cyst. Fibros. *16*, 483–487.

Deffert, C., Cachat, J., and Krause, K.H. (2014). Phagocyte NADPH oxidase, chronic granulomatous disease and mycobacterial infections. Cell. Microbiol. *16*, 1168–1178.

Del Porto, P., Cifani, N., Guarnieri, S., Di Domenico, E.G., Mariggiò, M.A., Spadaro, F., Guglietta, S., Anile, M., Venuta, F., Quattrucci, S., and Ascenzioni, F. (2011). Dysfunctional CFTR alters the bactericidal activity of human macrophages against *Pseudomonas aeruginosa*. PLoS ONE *6*, e19970.

Dewas, C., Dang, P.M., Gougerot-Pocidalo, M.A., and El-Benna, J. (2003). TNF-alpha induces phosphorylation of p47(phox) in human neutrophils: partial phosphorylation of p47phox is a common event of priming of human neutrophils by TNF-alpha and granulocyte-macrophage colony-stimulating factor. J. Immunol. *171*, 4392–4398.

Di, A., Brown, M.E., Deriy, L.V., Li, C., Szeto, F.L., Chen, Y., Huang, P., Tong, J., Naren, A.P., Bindokas, V., et al. (2006). CFTR regulates phagosome acidification in macrophages and alters bactericidal activity. Nat. Cell Biol. *8*, 933–944.

Donaldson, S.H., and Boucher, R.C. (2003). Update on pathogenesis of cystic fibrosis lung disease. Curr. Opin. Pulm. Med. *9*, 486–491.

Döring, G., and Gulbins, E. (2009). Cystic fibrosis and innate immunity: how chloride channel mutations provoke lung disease. Cell. Microbiol. *11*, 208–216.

Duranton, C., Rubera, I., Cougnon, M., Melis, N., Chargui, A., Mograbi, B., and Tauc, M. (2012). CFTR is involved in the fine tuning of intracellular redox status: physiological implications in cystic fibrosis. Am. J. Pathol. *181*, 1367–1377.

Ellett, F., Pase, L., Hayman, J.W., Andrianopoulos, A., and Lieschke, G.J. (2011). *mpeg1* promoter transgenes direct macrophage-lineage expression in zebrafish. Blood *117*, e49–e56.

Esther, C.R., Jr., Esserman, D.A., Gilligan, P., Kerr, A., and Noone, P.G. (2010). Chronic *Mycobacterium abscessus* infection and lung function decline in cystic fibrosis. J. Cyst. Fibros. *9*, 117–123.

Floto, R.A., Olivier, K.N., Saiman, L., Daley, C.L., Herrmann, J.L., Nick, J.A., Noone, P.G., Bilton, D., Corris, P., Gibson, R.L., et al.; US Cystic Fibrosis Foundation and European Cystic Fibrosis Society (2016). US Cystic Fibrosis Foundation and European Cystic Fibrosis Society consensus recommendations for the management of non-tuberculous mycobacteria in individuals with cystic fibrosis. Thorax *71* (*Suppl 1*), i1–i22.

Gabriel, S.E., Brigman, K.N., Koller, B.H., Boucher, R.C., and Stutts, M.J. (1994). Cystic fibrosis heterozygote resistance to cholera toxin in the cystic fibrosis mouse model. Science *266*, 107–109.

Gadsby, D.C., Vergani, P., and Csanády, L. (2006). The ABC protein turned chloride channel whose failure causes cystic fibrosis. Nature *440*, 477–483.

Hall, C., Flores, M.V., Storm, T., Crosier, K., and Crosier, P. (2007). The zebrafish lysozyme C promoter drives myeloid-specific expression in transgenic fish. BMC Dev. Biol. *7*, 42.

Harris, K.A., and Kenna, D.T. (2014). *Mycobacterium abscessus* infection in cystic fibrosis: molecular typing and clinical outcomes. J. Med. Microbiol. *63*, 1241–1246.

Hector, A., Griese, M., and Hartl, D. (2014). Oxidative stress in cystic fibrosis lung disease: an early event, but worth targeting? Eur. Respir. J. *44*, 17–19.

Howard, S.T., Rhoades, E., Recht, J., Pang, X., Alsup, A., Kolter, R., Lyons, C.R., and Byrd, T.F. (2006). Spontaneous reversion of *Mycobacterium abscessus* from a smooth to a rough morphotype is associated with reduced expression of glycopeptidolipid and reacquisition of an invasive phenotype. Microbiology *152*, 1581–1590.

Jönsson, B.E., Gilljam, M., Lindblad, A., Ridell, M., Wold, A.E., and Welinder-Olsson, C. (2007). Molecular epidemiology of *Mycobacterium abscessus*, with focus on cystic fibrosis. J. Clin. Microbiol. *45*, 1497–1504.

Kawakami, K., Takeda, H., Kawakami, N., Kobayashi, M., Matsuda, N., and Mishina, M. (2004). A transposon-mediated gene trap approach identifies developmentally regulated genes in zebrafish. Dev. Cell *7*, 133–144.

Kwan, K.M., Fujimoto, E., Grabher, C., Mangum, B.D., Hardy, M.E., Campbell, D.S., Parant, J.M., Yost, H.J., Kanki, J.P., and Chien, C.B. (2007). The Tol2kit: a multisite gateway-based construction kit for Tol2 transposon transgenesis constructs. Dev. Dyn. *236*, 3088–3099.

Lamason, R.L., Mohideen, M.A., Mest, J.R., Wong, A.C., Norton, H.L., Aros, M.C., Jurynec, M.J., Mao, X., Humphreville, V.R., Humbert, J.E., et al. (2005). SLC24A5, a putative cation exchanger, affects pigmentation in zebrafish and humans. Science *310*, 1782–1786.

Lavelle, G.M., White, M.M., Browne, N., McElvaney, N.G., and Reeves, E.P. (2016). Animal models of cystic fibrosis pathology: Phenotypic parallels and divergences. BioMed Res. Int. *2016*, 5258727.

Le Moigne, V., Rottman, M., Goulard, C., Barteau, B., Poncin, I., Soismier, N., Canaan, S., Pitard, B., Gaillard, J.L., and Herrmann, J.L. (2015). Bacterial phospholipases C as vaccine candidate antigens against cystic fibrosis respiratory pathogens: the *Mycobacterium abscessus model*. Vaccine *33*, 2118–2124.

Liu, F., Zhang, Z., Csanády, L., Gadsby, D.C., and Chen, J. (2017). Molecular structure of the human CFTR ion channel. Cell *169*, 85–95.e8.

Lyczak, J.B., Cannon, C.L., and Pier, G.B. (2002). Lung infections associated with cystic fibrosis. Clin. Microbiol. Rev. *15*, 194–222.

Malcolm, K.C., Caceres, S.M., Pohl, K., Poch, K.R., Bernut, A., Kremer, L., Bratton, D.L., Herrmann, J.L., and Nick, J.A. (2018). Neutrophil killing of *Mycobacterium abscessus* by intra- and extracellular mechanisms. PLoS ONE *13*, e0196120.

Meindl, R.S. (1987). Hypothesis: a selective advantage for cystic fibrosis heterozygotes. Am. J. Phys. Anthropol. *74*, 39–45.

Navis, A., and Bagnat, M. (2015). Loss of *cftr* function leads to pancreatic destruction in larval zebrafish. Dev. Biol. *399*, 237–248.

Navis, A., Marjoram, L., and Bagnat, M. (2013). Cftr controls lumen expansion and function of Kupffer's vesicle in zebrafish. Development *140*, 1703–1712.

Nessar, R., Cambau, E., Reyrat, J.M., Murray, A., and Gicquel, B. (2012). *Mycobacterium abscessus*: a new antibiotic nightmare. J. Antimicrob. Chemother. *67*, 810–818.

Nguyen-Chi, M., Laplace-Builhe, B., Travnickova, J., Luz-Crawford, P., Tejedor, G., Phan, Q.T., Duroux-Richard, I., Levraud, J.P., Kissa, K., Lutfalla, G., et al. (2015). Identification of polarized macrophage subsets in zebrafish. eLife *4*, e07288.

Nusslein-Volhard, C., and Dahm, R. (2002). Zebrafish: A Practical Approach (Oxford University Press).

Oberley-Deegan, R.E., Rebits, B.W., Weaver, M.R., Tollefson, A.K., Bai, X., McGibney, M., Ovrutsky, A.R., Chan, E.D., and Crapo, J.D. (2010). An oxidative environment promotes growth of *Mycobacterium abscessus*. Free Radic. Biol. Med. *49*, 1666–1673.

Ohga, S., Ikeuchi, K., Kadoya, R., Okada, K., Miyazaki, C., Suita, S., and Ueda, K. (1997). Intrapulmonary *Mycobacterium avium* infection as the first manifestation of chronic granulomatous disease. J. Infect. *34*, 147–150.

Olivier, K.N., Weber, D.J., Wallace, R.J., Jr., Faiz, A.R., Lee, J.H., Zhang, Y., Brown-Elliot, B.A., Handler, A., Wilson, R.W., Schechter, M.S., et al.; Nontuberculous Mycobacteria in Cystic Fibrosis Study Group (2003). Nontuberculous mycobacteria. I: multicenter prevalence study in cystic fibrosis. Am. J. Respir. Crit. Care Med. *167*, 828–834.

Orens, J.B., Estenne, M., Arcasoy, S., Conte, J.V., Corris, P., Egan, J.J., Egan, T., Keshavjee, S., Knoop, C., Kotloff, R., et al.; Pulmonary Scientific Council of the International Society for Heart and Lung Transplantation (2006). International guidelines for the selection of lung transplant candidates: 2006 update—a consensus report from the Pulmonary Scientific Council of the International Society for Heart and Lung Transplantation. J. Heart Lung Transplant. *25*, 745–755.

Parkins, M.D., and Floto, R.A. (2015). Emerging bacterial pathogens and changing concepts of bacterial pathogenesis in cystic fibrosis. J. Cyst. Fibros. *14*, 293–304.

Phennicie, R.T., Sullivan, M.J., Singer, J.T., Yoder, J.A., and Kim, C.H. (2010). Specific resistance to *Pseudomonas aeruginosa* infection in zebrafish is mediated by the cystic fibrosis transmembrane conductance regulator. Infect. Immun. *78*, 4542–4550.

Pier, G.B., Grout, M., Zaidi, T., Meluleni, G., Mueschenborn, S.S., Banting, G., Ratcliff, R., Evans, M.J., and Colledge, W.H. (1998). *Salmonella typhi* uses CFTR to enter intestinal epithelial cells. Nature *393*, 79–82.

Poolman, E.M., and Galvani, A.P. (2007). Evaluating candidate agents of selective pressure for cystic fibrosis. J. R. Soc. Interface *4*, 91–98.

Prajsnar, T.K., Cunliffe, V.T., Foster, S.J., and Renshaw, S.A. (2008). A novel vertebrate model of *Staphylococcus aureus* infection reveals phagocyte-dependent resistance of zebrafish to non-host specialized pathogens. Cell. Microbiol. *10*, 2312–2325.

Rada, B., and Leto, T.L. (2008). Oxidative innate immune defenses by Nox/Duox family NADPH oxidases. Contrib. Microbiol. *15*, 164–187.

Renshaw, S.A., and Trede, N.S. (2012). A model 450 million years in the making: zebrafish and vertebrate immunity. Dis. Model. Mech. *5*, 38–47.

Renshaw, S.A., Loynes, C.A., Trushell, D.M., Elworthy, S., Ingham, P.W., and Whyte, M.K. (2006). A transgenic zebrafish model of neutrophilic inflammation. Blood *108*, 3976–3978.

Rieber, N., Hector, A., Kuijpers, T., Roos, D., and Hartl, D. (2012). Current concepts of hyperinflammation in chronic granulomatous disease. Clin. Dev. Immunol. *2012*, 252460.

Roca, F.J., and Ramakrishnan, L. (2013). TNF dually mediates resistance and susceptibility to mycobacteria via mitochondrial reactive oxygen species. Cell *153*, 521–534.

Roux, A.L., Catherinot, E., Ripoll, F., Soismier, N., Macheras, E., Ravilly, S., Bellis, G., Vibet, M.A., Le Roux, E., Lemonnier, L., et al.; Jean-Louis Herrmann for the OMA Group (2009). Multicenter study of prevalence of nontuberculous mycobacteria in patients with cystic fibrosis in France. J. Clin. Microbiol. *47*, 4124–4128.

Roux, A.L., Viljoen, A., Bah, A., Simeone, R., Bernut, A., Laencina, L., Deramaudt, T., Rottman, M., Gaillard, J.L., Majlessi, L., et al. (2016). The distinct fate of smooth and rough *Mycobacterium abscessus* variants inside macrophages. Open Biol. *6*, 160185.

Snapper, S.B., Melton, R.E., Mustafa, S., Kieser, T., and Jacobs, W.R. (1990). Isolation and characterization of efficient plasmid transformation mutants of *Mycobacterium smegmatis*. Mol. Microbiol. *4*, 1911–1919.

Stinear, T.P., Seemann, T., Harrison, P.F., Jenkin, G.A., Davies, J.K., Johnson, P.D., Abdellah, Z., Arrowsmith, C., Chillingworth, T., Churcher, C., et al. (2008). Insights from the complete genome sequence of *Mycobacterium marinum* on the evolution of *Mycobacterium tuberculosis*. Genome Res. *18*, 729–741.

Tomashefski, J.F., Jr., Stern, R.C., Demko, C.A., and Doershuk, C.F. (1996). Nontuberculous mycobacteria in cystic fibrosis. An autopsy study. Am. J. Respir. Crit. Care Med. *154*, 523–528.

Torraca, V., Masud, S., Spaink, H.P., and Meijer, A.H. (2014). Macrophage-pathogen interactions in infectious diseases: new therapeutic insights from the zebrafish host model. Dis. Model. Mech. *7*, 785–797.

Vergunst, A.C., Meijer, A.H., Renshaw, S.A., and O'Callaghan, D. (2010). *Burkholderia cenocepacia* creates an intramacrophage replication niche in zebrafish embryos, followed by bacterial dissemination and establishment of systemic infection. Infect. Immun. *78*, 1495–1508.

Warnatsch, A., Tsourouktsoglou, T.D., Branzk, N., Wang, Q., Reincke, S., Herbst, S., Gutierrez, M., and Papayannopoulos, V. (2017). Reactive oxygen species localization programs inflammation to clear microbes of different size. Immunity *46*, 421–432.

Weaver, C.J., Leung, Y.F., and Suter, D.M. (2016). Expression dynamics of NADPH oxidases during early zebrafish development. J. Comp. Neurol. *524*, 2130–2141.

Weening, R.S., De Boer, M., Kuijpers, T.W., Neefjes, V.M., Hack, W.W., and Roos, D. (2000). Point mutations in the promoter region of the *CYBB* gene leading to mild chronic granulomatous disease. Clin. Exp. Immunol. *122*, 410–417.

Zhang, Z., and Chen, J. (2016). Atomic structure of the cystic fibrosis transmembrane conductance regulator. Cell *167*, 1586–1597.e9.

STAR★METHODS

KEY RESOURCES TABLE

REAGENT or RESOURCE	SOURCE	IDENTIFIER
Experimental Model: Bacterial Strains		
Mycobacterium abscessus sensu stricto, strain CIP104536^T, smooth	Laboratoire de Référence des Mycobactéries (IP, France)	ATCC19977^T
Mycobacterium abscessus sensu stricto, strain CIP104536^T, rough	Laboratoire de Référence des Mycobactéries (IP, France)	ATCC19977^T
Mycobacterium abscessus subsp. bolletii, CIP108541^T, smooth	Laboratoire de Référence des Mycobactéries (IP, France)	CIP108541^T
Mycobacterium abscessus subsp. bolletii, CIP108541^T, rough	Bernut et al., 2016b	CIP108541^T
Mycobacterium abscessus subsp. massiliense, CIP108297^T, smooth	Laboratoire de Référence des Mycobactéries (IP, France)	CIP108297^T
Mycobacterium abscessus subsp. massiliense, CIP108297^T, rough	Laboratoire de Référence des Mycobactéries (IP, France)	CIP108297^T
Mycobacterium marinum strain M	Stinear et al., 2008	ATCC BAA-535
Mycobacterium chelonae strain A6	Laboratoire de Référence des Mycobactéries (IP, France)	N/A
Mycobacterium smegmatis mc^{2}155	Snapper et al., 1990	N/A
Experimental Model: Zebrafish lines		
golden mutant	Lamason et al., 2005	N/A
cftr^{pd1049} mutant	Navis et al., 2013	N/A
gBAC(cftr-RFP)pd1042	Navis et al., 2013	N/A
Tg(mpx:GFP)i114	Renshaw et al., 2006	N/A
Tg(LysC_DSred)nz5	Hall et al., 2007	N/A
Tg(mpeg1:NLSmclover)sh436	This study	N/A
Tg(mpeg1:mCherry-F)ump2	Bernut et al., 2014	N/A
Tg(tnfα:GFP-F)ump5	Nguyen-Chi et al., 2015	N/A
Chemicals, Peptides, and Recombinant Proteins		
Hygromycin B	Sigma-Aldrich	Cat# H3274
Tricaine	Sigma-Aldrich	Cat# E10521
Difco Middlebrook 7H9 Broth	Thermo Fisher Scientific	Cat# DF0713-17-9
Middlebrook OADC Growth Supplement	Sigma-Aldrich	Cat# M0678
Tween-80	Sigma-Aldrich	Cat# P1754
f-Met-Leu-Phe (fMLP)	Sigma-Aldrich	Cat# F3506
Acridine Orange	Invitrogen	Cat# 93001
CellROX Deep Red	Invitrogen	*Cat# C10422*
LR Clonase II Plus	Invitrogen	*Cat# 12538*
SuperScript IV First-Strand Synthesis System	Invitrogen	*Cat# 18091050*
LightCycler® 480 SYBR Green I Master	Roche	Cat# 04887352001
Recombinant DNA		
pTEC15	Addgene	Cat# 30174
pTEC19	Addgene	Cat# 30178
pTEC27	Addgene	Cat# 30182
pcDNA3.1-Clover-mRuby2	Addgene	Cat# 49089
Gateway pDONR221 Vector	Invitrogen	Cat# 12536017
pCSTP	Kawakami et al., 2004	N/A

(*Continued on next page*)

Continued

REAGENT or RESOURCE	SOURCE	IDENTIFIER
Critical Commercial Assays		
Nucleospin RNAII kit	Macherey-Nagel	Cat# 740955
Oligonucleotides		
Primers used in this study are listed in Table S1		
Software and Algorithms		
Prism 7.0	Graphpad	https://www.graphpad.com/; RRID:SCR_002798
R 3.5.0	R core team	http://www.r-project.org; RRID:SCR_001905
ImageJ	NIH	https://imagej.nih.gov/ij/; RRID:SCR_003070
TIA Software	Thermo Scientific Tecnai	https://www.fei.com
Volocity 6.3	PerkinElmer Life and Analytical Sciences, Cambridge, UK	http://www.perkinelmer.com/fr/lab-products-and-services/resources/whats-new-volocity-6-3.html RRID:SCR_002668
LAS-AF	Leica Microsystems	https://www.leica-microsystems.com/products/microscope-software.html
Zen (Blue edition)	Zeiss	https://www.zeiss.com/microscopy/int/products/microscope-software/zen.html
LightCycler® 480 Software	Roche	https://lifescience.roche.com/en_gb/products/lightcycler14301-480-software-version-15.html

CONTACT FOR REAGENT AND RESOURCE SHARING

Further information and requests for resources and reagents should be directed to and will be fulfilled by the Lead Contact, Laurent Kremer (laurent.kremer@irim.cnrs.fr).

EXPERIMENTAL MODELS AND SUBJECT DETAILS

Bacterial Strains

Mycobacterial strains carrying pTEC15 (Addgene, plasmid 30174), pTEC27 (Addgene, plasmid 30182) or pTEC19 (Addgene, plasmid 30178) that express green fluorescent protein (Wasabi), red fluorescent protein (tdTomato) or bright far-red fluorescent protein (E2-Crimson), respectively, were grown under hygromycin B selection in Middlebrook 7H9 supplemented with oleic acid, albumin, dextrose, catalase (OADC), and 0.05% Tween-80. To prepare heat-killed Mabs, bacteria were incubated at 80°C for 20 min.

Zebrafish Husbandry and Ethic statements

Experimental procedures were performed using the golden mutant (Lamason et al., 2005), the $cftr^{pd1049}$ mutant (Navis et al., 2013), the transgenic lines Tg(mpx:GFP)i114 (Renshaw et al., 2006) and Tg(LysC_DSred)nz5 (Hall et al., 2007) to visualize neutrophils; Tg(mpeg1:NLSclover)sh436 and Tg(mpeg1:mCherry-F)ump2 (Bernut et al., 2014) to visualize MΦ ; *gBAC(cftr-RFP)pd1042* (Navis et al., 2013) to visualize cftr expression, and Tg(tnfα:GFP-F)ump5 (Nguyen-Chi et al., 2015) to visualize tnf-α expression. ZF were raised and maintained according to standard protocols (Nusslein-Volhard and Dahm, 2002). Eggs were obtained from pairs of adult fish by natural spawning and raised at 28.5°C in tank water. The ZF husbandry and all ZF experiments described in the present study were conducted in accordance with guidelines from the UK Home Office (Bateson Centre, University of Sheffield) and in compliance with the European Union guidelines for handling of laboratory animals (CNRS, Montpellier) and were approved by the Direction Sanitaire et Vétérinaire de l'Hérault et Comité d'Ethique pour l'Expérimentation Animale de la région Languedoc Roussillon under the reference CEEA-LR-1145 (Montpellier experiments).

METHOD DETAILS

Creation of theTg(mpeg1:NLSmClover) transgenic line

The Tol2kit multisite Gateway-based transposon system (Kwan et al., 2007) was used to generated a construct from which a stable transgenic line was raised. mClover was amplified from pcDNA3.1-Clover-mRuby2, a gift from Kurt Beam (Addgene, plasmid 49089), using forward forward primer 5′-GGGGACAAGTTTGTACAAAAAAGGCTCAATGGCTCCAAAGAAGAAGCGTAAGGTA-3′ and reverse primer 5′-GGGGACCACTTTGTACAAGAAAGCTGGGTCTACTTGTACAGCTCGTCCA-3′ and cloned into pDONR 221 donor vector (Invitrogen) to produce pME-NLSmClover. An LR Clonase II Plus (Invitrogen) Gateway reaction was performed with p5E-mpeg1 (Ellett et al., 2011), pME-NLSmClover and p3E-polyA inserted into pDestTol2pA2 destination vector (Kwan et al., 2007) to

produce *mpeg1:NLSmClover* construct. The *tol2*-transposase mRNA was synthesized from pCSTP (Kawakami et al., 2004) and co-injected with the *mpeg1:NLSmClover* construct into one-cell-stage ZF embryos to create the *Tg(mpeg1:NLSmClover)sh436* transgenic line harboring green fluorescent macrophages.

Morpholino injection

MO were purchased from Gene Tools. *cftr* splice-blocking morpholino targeting *cftr* (ZFIN, ZDB-GENE-050517-20) (5′-GACA CATTTTGGACACTCACACCAA-3′) was injected into one-cell-stage zebrafish embryos (1 mM, 2 nl). The efficiency of gene knockdown was confirmed by RT-PCR and sequencing with the following primers for both sides of the MO target sequence (forward and reverse): CCTGTGGAGGATGCCAACTGCC and TGCATGCCCAGGTGGTGCAG. Total RNA from 2 dpf embryos (pools of 10 each) was isolated and purified with Nucleospin RNAII kit (Macherey-Nagel), following the manufacturer's instructions. The SuperScript IV reverse transcriptase (Invitrogen) was used to synthesize first-strand cDNA with oligo(dT) primer from 1 μg of total RNA at 50°C for 50 min. MO for *nox2* (*cybb*, ZFIN, ZDB-GENE-040426-1380) knockdown (5′-CATAATCCCGATAGCTTACGATAAC-3′) was prepared and injected as described earlier (Roca and Ramakrishnan, 2013). A standard control-MO was included as a negative control in all experiments.

Zebrafish infection

Bacterial inoculate were prepared for infection challenges in ZF embryos/larvae, according to procedures described earlier (Bernut et al., 2015). Briefly, systemic infections were carried out by the injection of single-cell suspensions of known titer (100-200 colony-forming units) into the caudal vein of 30 hpf embryos. For leucocyte mobilization assays, mycobacteria were locally injected into the hindbrain ventricle at 30 hpf, the otic vesicle or the muscle compartment of 3 dpf larvae. The number of animals used for each procedure was guided by pilot experiments or by past results (Bernut et al., 2016a; Bernut et al., 2014).

qRT-PCR

Total RNA was prepared and first-strand cDNA synthesized. Real-time RT-PCRs were performed with an LightCycler® 480 system instrument using LightCycler® 480 SYBR Green I Master (Roche) and gene expressions were detected with gene-specific primers listed in Table S1. Reaction mixtures were incubated for 5 min at 95°C, followed by 45 cycles of 5 s at 95°C, 20 s at 65°C, and finally 10 s at 95°C. Each experiment was run in triplicate. qRT-PCR data are analyzed with the LightCycler® 480 Software, normalized to the housekeeping gene ef1α and calculated using the ΔΔCt method.

Epifluorescence, Confocal Microscopy and Imaging

To quantify bacterial loads, granulomas (defined as cellular aggregates comprising at least 10 infected cells), cords, leukocyte mobilization, and evaluate oxidative stress, infected larvae were tricaine-anesthetized and mounted in 0.8% low melting point agarose with 0.016% tricaine for real-time microscopy observations. To evaluate intracellular mycobacterial growth, phagocytosis, cellular mortality, and granuloma organization, infected animals were tricaine-anesthetized, fixed overnight at 4°C in 4% paraformaldehyde in PBS, washed twice in PBS and then transferred gradually from PBS to 50% glycerol for microscopy observation.

Epifluorescence microscopy was performed using a Zeiss Plan Neo Fluor Z 1x/0.25 FWD objective and equipped with an Axiocam503 monochrome (Zeiss) camera. Pictures were taken and processed using ZEN 2 (blue edition). Confocal microscopy was performed using a Spinning disc confocal Perkin Elmer Ultraview VoX inverted Olympus IX81 with a UplanSAPO 20x/0.8 and a UplanSAPO 40x/1.3 oil objective, equipped with a Hamamatsu C9100-50 EM-CCD camera or with a Leica SPE upright microscope with a ACS APO 40x/1.15 oil objective. Images were captured and processed using the Velocity or LASAFS softwares.

Transmission Electron Microscopy

For TEM, ZF larvae were tricaine-anesthetized and fixed overnight at 4°C in 2.5% glutaraldehyde in 0.1M phosphate buffer. After a post-fixation in 1% osmic acid 1 hr at 4°C and 0.5% tanic acid for 30 min at 4°C, animals were dehydrated in successive ethanol baths, infiltrated with mixes of epon 812/propylene oxide, embedded in epon 812 resin, and then polymerized at 60°C for 48 hr. Ultrathin sections were cut with a Reichert Ultracut ultramicrotome (Leica) and collected on nickel grids. Finally, the grids were stained 20 min in 2% uranyle acetate and 3 min in 3% lead citrate, then examined on a Tecnai G2 F20 (200kV, FEG) electron microscope. Images were captured using TIA imaging software.

Neutrophils Recruitment Assay

Neutrophil mobilization was elicited through injection of 300 nM f-Met-Leu-Phe (fMLP) chemoattractant into the otic cavity of 3 dpf larvae (Bernut et al., 2016a) and counting the recruited neutrophils at the injection site using fluorescence microscopy.

Cell Death and Reactive Oxygen Species Detection

Leucocyte killing in ZF was detected using Acridine Orange (AO), as previously described (Bernut et al., 2014). Living embryos were soaked in 10 mg/ml AO in fish water for 30 min at 28.5°C, followed by two washes, then replaced at 28.5°C until observation and

analyzed by confocal microscopy. For ROS detection, living larvae were soaked in 5 μM CellROX Deep Red Reagent in PBS for 30 min at 28.5°C, rinsed twice with PBS and directly prepared for confocal microscopy observations.

QUANTIFICATION AND STATISTICAL ANALYSIS

Statistical analysis (Prism 7.0; GraphPad Software or R 3.5.0) was performed using χ^2Fisher's exact test, two-tailed unpaired Student's t test for comparisons between two groups and one-way or two-way ANOVA (with appropriate post-test adjustment) for other data. Mantel-Cox Log-rank test was used to compare survival curves. All data are plotted as average of two or three independent experiments. All error bars indicate standard errors of means (SEM). ns, not significant ($p \geq 0.05$); *$p < 0.05$; **$p < 0.01$; ***$p < 0.001$; ****$p < 0.0001$.

Cell Reports, Volume *26*

Supplemental Information

CFTR Protects against *Mycobacterium abscessus* Infection by Fine-Tuning Host Oxidative Defenses

Audrey Bernut, Christian Dupont, Nikolay V. Ogryzko, Aymeric Neyret, Jean-Louis Herrmann, R. Andres Floto, Stephen A. Renshaw, and Laurent Kremer

Supplemental Table 1. Primers used in this study, related to STAR Methods.

Designation	Sequences	References
ef1a (for qPCR)	Forward : 5′-TCTGTTACCTGGCAAAGGG-3’	Bernut et al, 2016a
	Reverse : 5′-TTCAGTTTGTCCAACACCCA-3’	Bernut et al, 2016a
cxcl8a (for qPCR)	Forward : 5′-CCTGGCATTTCTGACCATCAT-3’	Bernut et al, 2016a
	Reverse : 5′-GATCTCCTGTCCAGTTGTCAT-3’	Bernut et al, 2016a
Tnfa (for qPCR)	Forward : 5′-TTCACGCTCCATAAGACCCA-3’	Bernut et al, 2016a
	Reverse : 5′-CCGTAGGATTCAGAAAAGCG-3’	Bernut et al, 2016a
nox2 (for qPCR)	Forward : 5′-CTTTCGTTATGAAGCGGTGATG-3’	Weaver et al, 2016
	Reverse : 5′-GGTTCTCCTGGACGTGTTTAT-3’	Weaver et al, 2016
Control-MO	5'-CCTCTTACCTCAGTTACAATTTATA-3'	Gene Tools
cftr-MO	5'-GACACATTTTGGACACTCACACCAA-3'	This study
nox2-MO	5'-CATAATCCCGATAGCTTACGATAAC-3'	Roca and Ramakrishnan, 2013
cftr for MO screen efficiency	Forward : 5′-CCTGTGGAGGATGCCAACTGCC-3’	This study
	Reverse : 5′-TGCATGCCCAGGTGGTGCAG-3’	This study
pME NLS Clover	Forward : 5’-GGGGACAAGTTTGTACAAAAAAAGGCTCAATGGCTCCAAAGAAGAAGCGTAAGGTA-3’	This study
	Reverse : 5’-GGGGACCACTTTGTACAAGAAAGCTGGGTCTACTTGTACAGCTCGTCCA-3’	This study

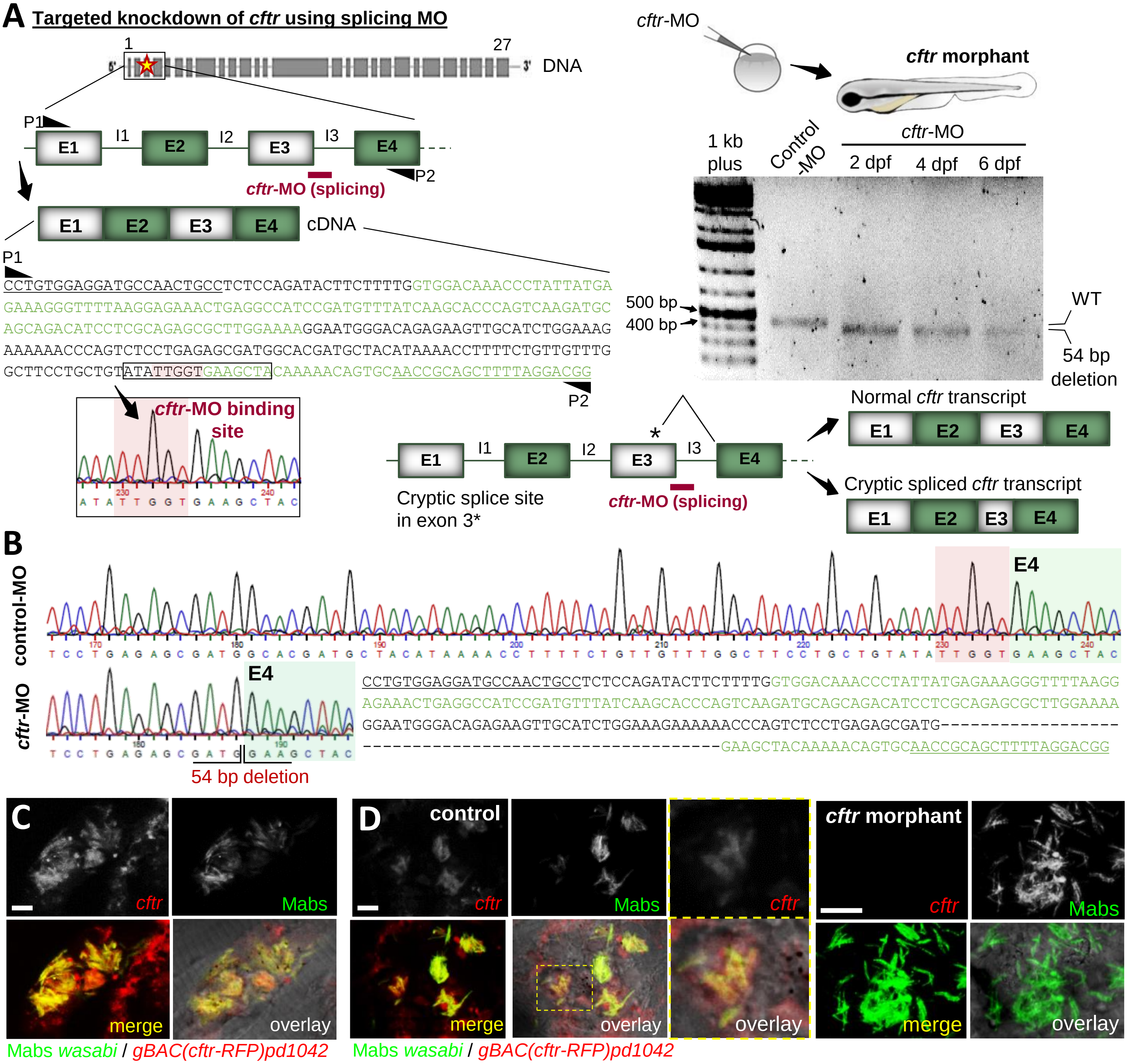

Figure S1. Targeted knockdown of *cftr* using splicing morpholino in ZF embryos, related to Figure 1
(A-B) A splice targeting morpholino was designed against exon3-intron3 boundary within the *cftr* gene. (A) Schematic representation and nucleotide sequence corresponding to exon 1 through exon 4 of the ZF *cftr* gene with the splice morpholino *cftr*-MO binding site and locations of primers used to amplify the region surrounding exon 3 (P1/P2) (left panel). Altered splicing in *cftr* MO injected-embryos is verified by RT-PCR (right panel). *cftr* specific products were amplified from RNA isolated from whole embryos at 2, 4 and 6 dpf. The *cftr*-MO effectively blocks the splice donor site at the exon3-intron3 boundary, giving rise to an amplicon with a reduce size as compared to the size of the normal *cftr* transcript, suggesting the existence of a cryptic splice site within exon 3.
(B) Comparison of the sequences shows that *cftr*-MO blocks normal splicing resulting in 54pb deletion in exon 3, thus confirming the efficacy and specificity of this morpholino.
(C) Confocal images showing the representative *cftr* expression in Mabs S- induced granuloma in control-MO-injected *gBAC(cftr-RFP)pd104* embryos infected with Mabs expressing Wasabi (5 dpi). Scale bar, 5 µm.
(D) Confocal images showing the *cftr* expression in infected phagocytes in a control or a *cftr* morphant *gBAC(cftr-RFP)pd104* embryos infected with Mabs S expressing Wasabi (5 dpi). Scale bars, 5 µm.

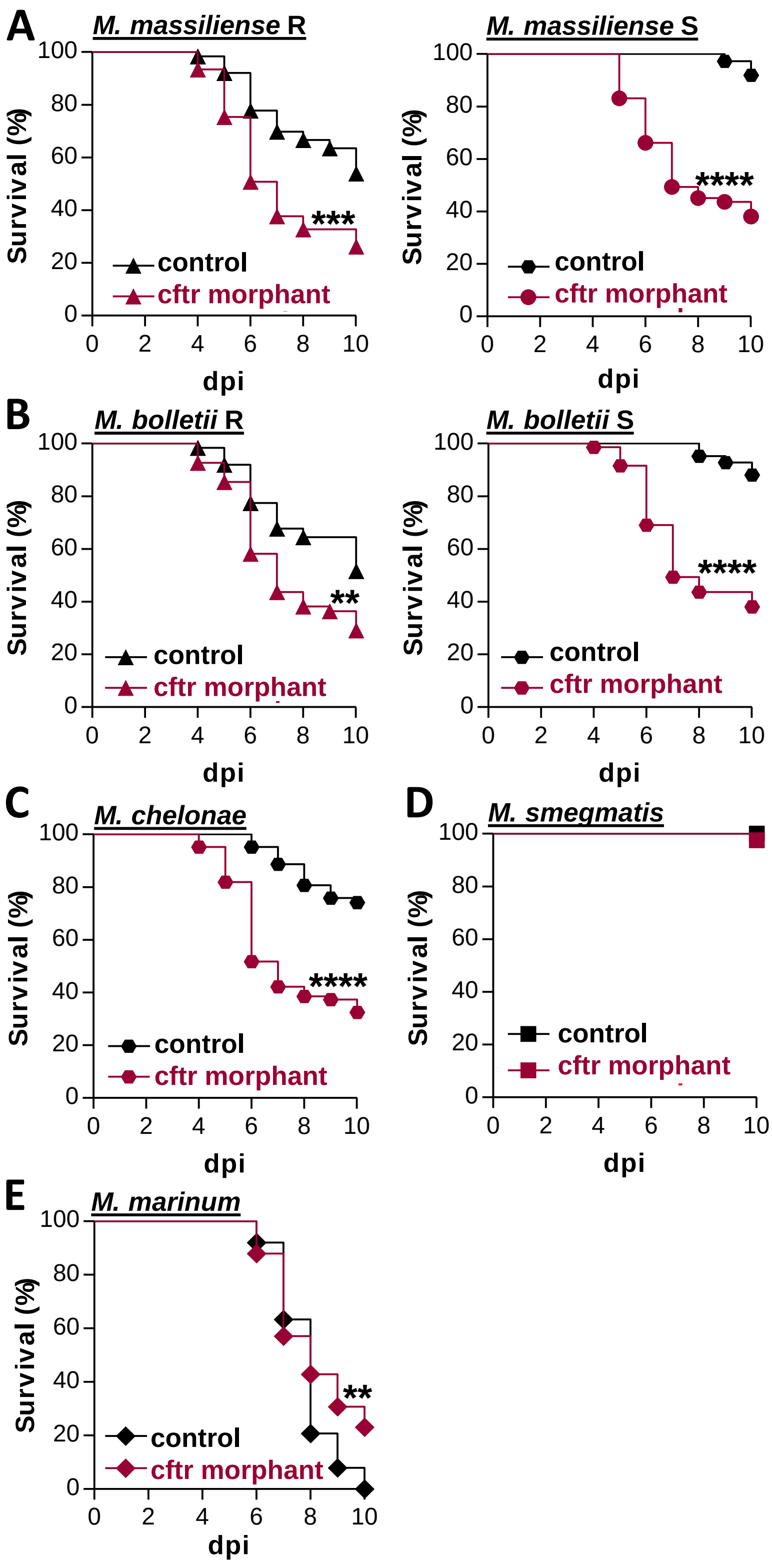

Figure S2. *Cftr* MO knockdown results in increased susceptibility to NTM, related to Figure 1

(A-E) Control embryos or *cftr* morphants were intravenously infected with ≈150 CFU of various mycobacteria expressing tdTomato. Data are plotted as percentage of surviving animal on each day (n=40, average of two independent experiments, Mantel-Cox Log-rank test).

(A) Survival analysis of embryos infected with either R (top graph) or S (bottom graph) variants of *M. massiliense*.

(B) Survival analysis of embryos infected with either R (top graph) or S (bottom graph) variants of *M. bolletii*.

(C) Survival analysis of *M. chelonae*-infected larvae.

(D) Survival analysis of *M. smegmatis*-infected larvae.

(E) Survival analysis of *M. marinum*-infected larvae.

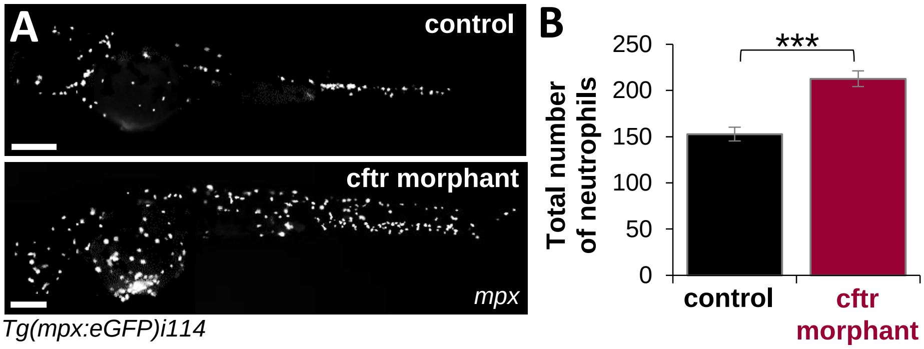

Figure S3. ***cftr*** **knockdown promotes a basal hyperneutrophilia, related to Figure 4**

(A) Representative fluorescence microscopy images of control embryos *versus cftr* morphants *Tg(mpx:eGFP)i114* embryos at 2 dpf. Scale bars, 200 µm.

(B) Basal number of neutrophils in whole embryos at 2 dpf. Graphs represent the mean ±SEM of two independent experiments (n=10). Significance was assessed by two-tailed unpaired Student's t test comparing both infected embryos per category.

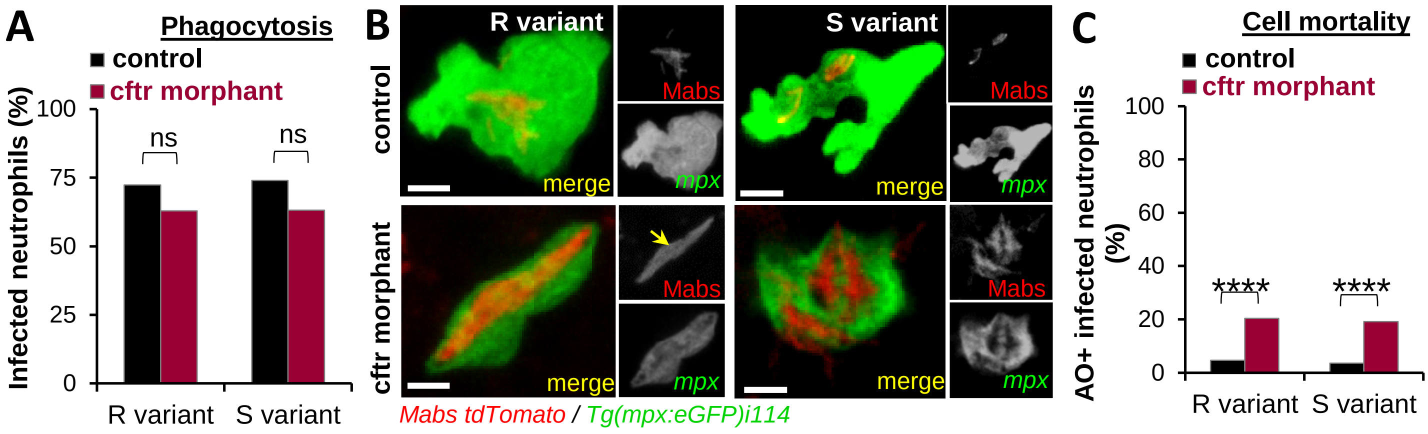

Figure S4, *cftr* knock-down inhibits *M. abscessus* killing inside neutrophils, related to Figure 4

(A) Control or *cftr* morphants *Tg(mpx:eGFP)i114* larvae were infected into the muscle with ≈100 CFU Mabs R or S expressing tdTomato. Proportion of infected neutrophils analyzed using confocal microscopy and recruited in the infected site at 4 hpi. Data are plotted as mean ± SEM from two independent experiments (n=20).

(B) Control or *cftr* morphants *Tg(mpx:eGFP)i114* embryos were *iv* infected with Mabs R or S expressing dtTomato. Confocal images showing infected neutrophils. While WT-neutrophils efficiently contain intracellular bacilli, CF-neutrophils appear overloaded with Mabs. Arrow indicates intracellular Mabs R cording. Scale bar, 2 µm.

(C) Control or *cftr* morphants *Tg(LysC_DSred)nz5* embryos were *iv* infected with ≈100 CFU Mabs R or S expressing E2-Crimson and stained for dead cells using acridine orange (AO). Proportion of dead infected neutrophils was evaluated in the CHT at 2 dpi using confocal microscopy. Data are plotted as mean ± SEM from two independent experiments (n=20).

Significance was assessed by Fisher's exact test (A and C).

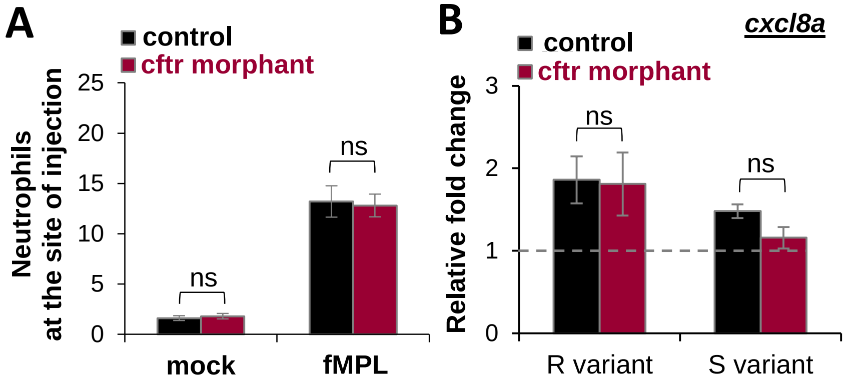

Figure S5. *cftr* knock-down does not impair the neutrophil chemotaxis functions, related to Figure 4
(A) Mean ± SEM number of recruited neutrophils into the otic cavity in response to mock or fMLP injection in control and *cftr* morphants *Tg(mpx:eGFP)i114* larvae monitored using confocal microscopy at 3 hpi (two independent experiments, n=14).
(B) qRT-PCR measurement for whole embryos 4 dpi after intravenous infection with ≈150CFU Mabs R or S and plotted as fold increase over mock injection for *cxcl8a*. Mean relative ± SEM gene expression of three independent replicates.
Significance was assessed by two-tailed unpaired Student’s t test comparing both infected embryos per category (A and B).

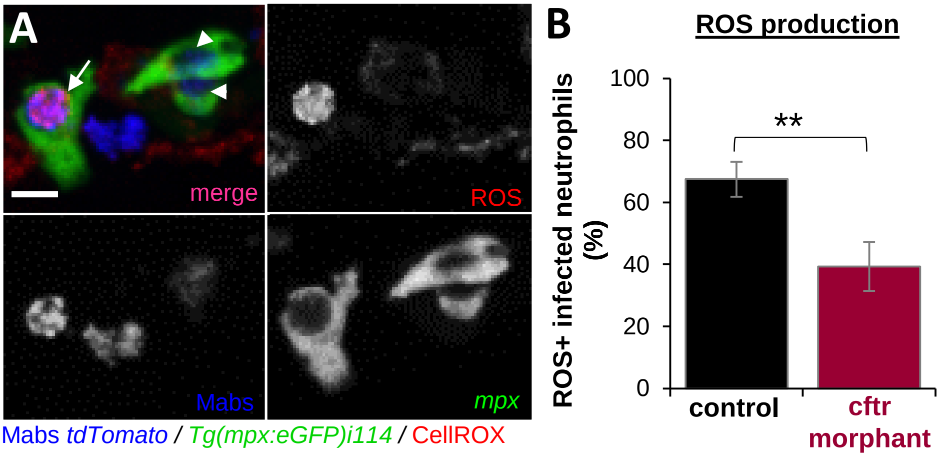

Figure S6, *cftr* knock-down altered neutrophil ROS production, related to Figure 6

(A-B) Control or *cftr* morphants *Tg(mpx:eGFP)i114* larvae were infected with ≈150 CFU Mabs expressing dtTomato into the muscle and stained for ROS production using CellRox Deep red.

(A) Confocal imaging showing both ROS$^+$ (arrow) or ROS$^-$ (arrow heads) -producing infected neutrophils (arrow) at the infected site. Scale bar, 5 µm.

(B) Proportion of ROS-producing infected neutrophils were evaluated at 4 hpi using confocal microscopy (from two independent experiments (n=16)). Significance was assessed by Fisher's exact test.

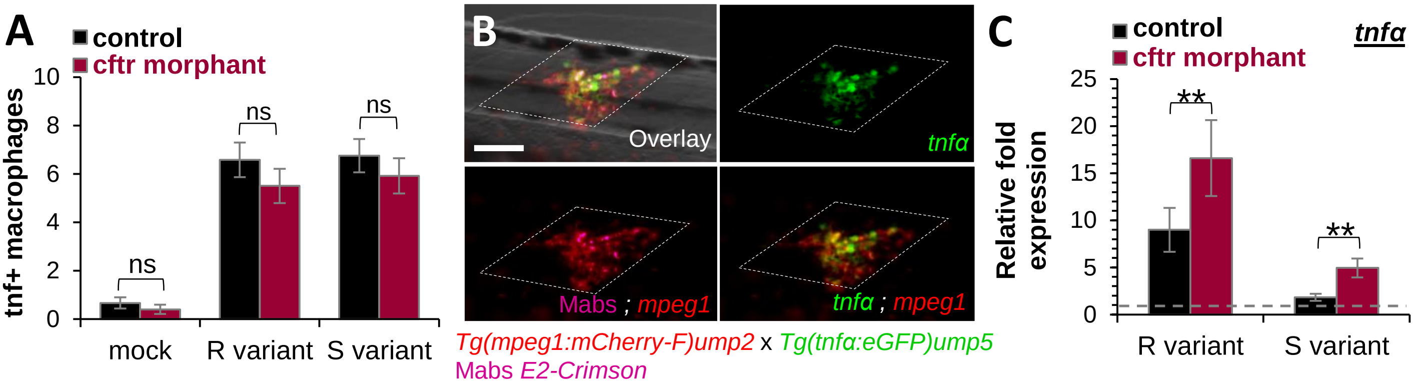

Figure S7. *M. abscessus* infection triggers hyperinflammation in the absence of CFTR, related to Figure 6

(A-B) Double transgenic larvae *Tg*(*tnfα*:*eGFP-F)ump5* / *Tg*(*mpeg1*:*mCherry-F*)*ump2)* were infected into the muscle with ≈100 Mabs R or S variants expressing E2-Crimson or with PBS (mock).

(A) Quantification of *tnf* $^{+}$ MΦ per infected larvae after 2 hpi. The data are representative of two experiments.

(B) Microscopy showing the representative expression of GFP^{+} cells at 2 hpi at the injection site. Scale bar, 70 µm.

(C) qRT-PCR measurement for whole Mabs R- or S-infected embryos 4 dpi (≈150 CFU) and plotted as fold increase over mock injection for *tnfα*. Mean relative ± SEM gene expression of three independent replicates. Significance was assessed by two-tailed unpaired Student's t test comparing both infected embryos per category (A and C).
