# Supplementary material for: CFTR Protects against Mycobacterium abscessus Infection by Fine-Tuning Host Oxidative Defenses
Source: Cell Rep. Author manuscript; Available in PMC 2025 Nov 18. (PMC7618368; doi:10.1016/j.celrep.2019.01.071)
Supplement: Supplementary Material — Supplemental Information includes one table and seven figures and can be found with this article online at https://doi.org/10.1016/j.celrep.2019.01.071. [file EMS209705-supplement-Supplementary_Material.zip › 1-s2.0-S2211124719301007-mmc1.pdf]

Cell Reports, Volume *26*

Supplemental Information

CFTR Protects against *Mycobacterium abscessus* Infection by Fine-Tuning Host Oxidative Defenses

Audrey Bernut, Christian Dupont, Nikolay V. Ogryzko, Aymeric Neyret, Jean-Louis Herrmann, R. Andres Floto, Stephen A. Renshaw, and Laurent Kremer

Supplemental Table 1. Primers used in this study, related to STAR Methods.

Designation	Sequences	References
ef1a (for qPCR)	Forward : 5′-TCTGTTACCTGGCAAAGGG-3’	Bernut et al, 2016a
	Reverse : 5′-TTCAGTTTGTCCAACACCCA-3’	Bernut et al, 2016a
cxcl8a (for qPCR)	Forward : 5′-CCTGGCATTTCTGACCATCAT-3’	Bernut et al, 2016a
	Reverse : 5′-GATCTCCTGTCCAGTTGTCAT-3’	Bernut et al, 2016a
Tnfa (for qPCR)	Forward : 5′-TTCACGCTCCATAAGACCCA-3’	Bernut et al, 2016a
	Reverse : 5′-CCGTAGGATTCAGAAAAGCG-3’	Bernut et al, 2016a
nox2 (for qPCR)	Forward : 5′-CTTTCGTTATGAAGCGGTGATG-3’	Weaver et al, 2016
	Reverse : 5′-GGTTCTCCTGGACGTGTTTAT-3’	Weaver et al, 2016
Control-MO	5'-CCTCTTACCTCAGTTACAATTTATA-3'	Gene Tools
cftr-MO	5'-GACACATTTTGGACACTCACACCAA-3'	This study
nox2-MO	5'-CATAATCCCGATAGCTTACGATAAC-3'	Roca and Ramakrishnan, 2013
cftr for MO screen efficiency	Forward : 5′-CCTGTGGAGGATGCCAACTGCC-3’	This study
	Reverse : 5′-TGCATGCCCAGGTGGTGCAG-3’	This study
pME NLS Clover	Forward : 5’-GGGGACAAGTTTGTACAAAAAAAGGCTCAATGGCTCCAAAGAAGAAGCGTAAGGTA-3’	This study
	Reverse : 5’-GGGGACCACTTTGTACAAGAAAGCTGGGTCTACTTGTACAGCTCGTCCA-3’	This study

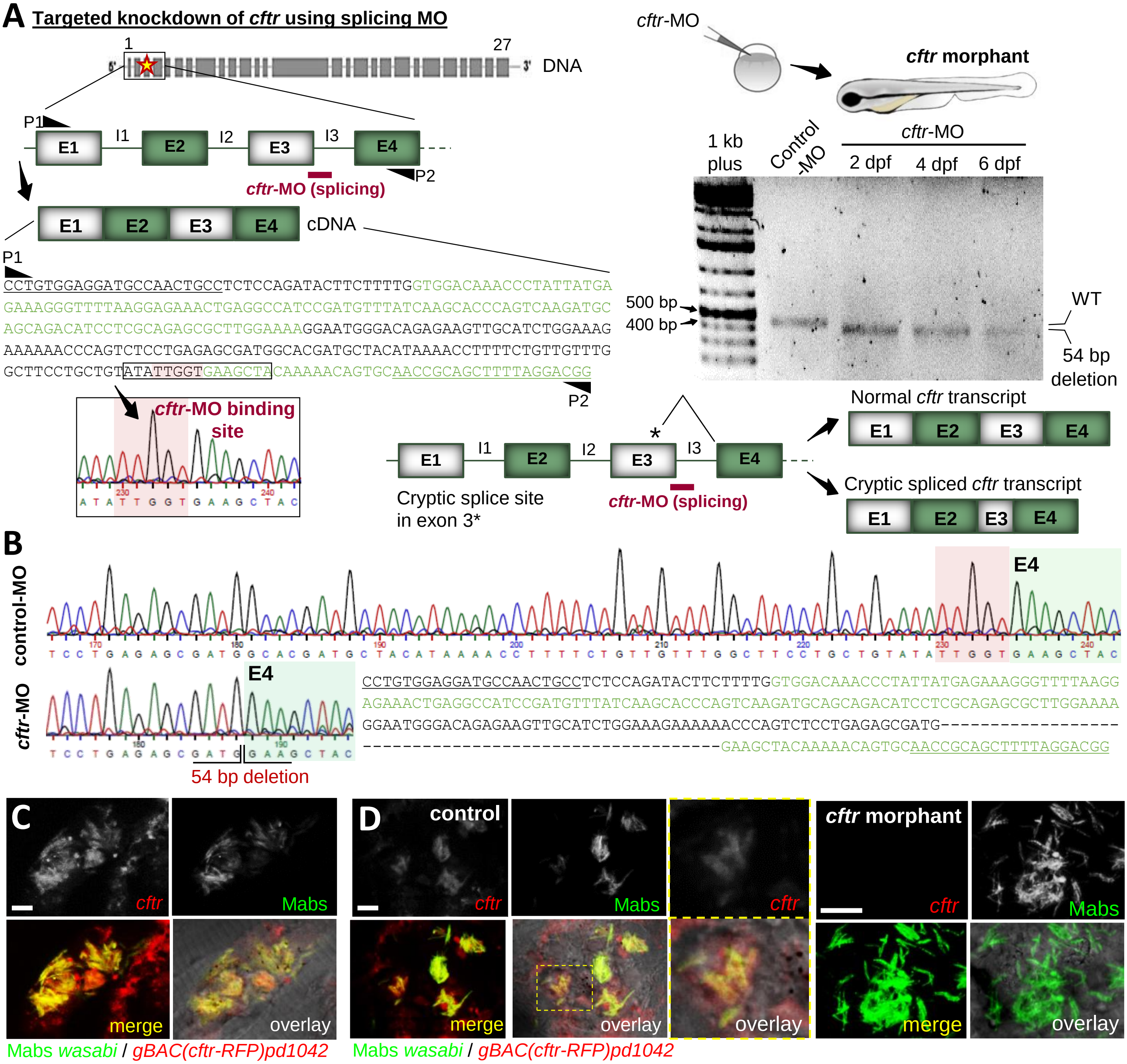

Figure S1. Targeted knockdown of *cftr* using splicing morpholino in ZF embryos, related to Figure 1
(A-B) A splice targeting morpholino was designed against exon3-intron3 boundary within the *cftr* gene. (A) Schematic representation and nucleotide sequence corresponding to exon 1 through exon 4 of the ZF *cftr* gene with the splice morpholino *cftr*-MO binding site and locations of primers used to amplify the region surrounding exon 3 (P1/P2) (left panel). Altered splicing in *cftr* MO injected-embryos is verified by RT-PCR (right panel). *cftr* specific products were amplified from RNA isolated from whole embryos at 2, 4 and 6 dpf. The *cftr*-MO effectively blocks the splice donor site at the exon3-intron3 boundary, giving rise to an amplicon with a reduce size as compared to the size of the normal *cftr* transcript, suggesting the existence of a cryptic splice site within exon 3.
(B) Comparison of the sequences shows that *cftr*-MO blocks normal splicing resulting in 54pb deletion in exon 3, thus confirming the efficacy and specificity of this morpholino.
(C) Confocal images showing the representative *cftr* expression in Mabs S- induced granuloma in control-MO-injected *gBAC(cftr-RFP)pd104* embryos infected with Mabs expressing Wasabi (5 dpi). Scale bar, 5 µm.
(D) Confocal images showing the *cftr* expression in infected phagocytes in a control or a *cftr* morphant *gBAC(cftr-RFP)pd104* embryos infected with Mabs S expressing Wasabi (5 dpi). Scale bars, 5 µm.

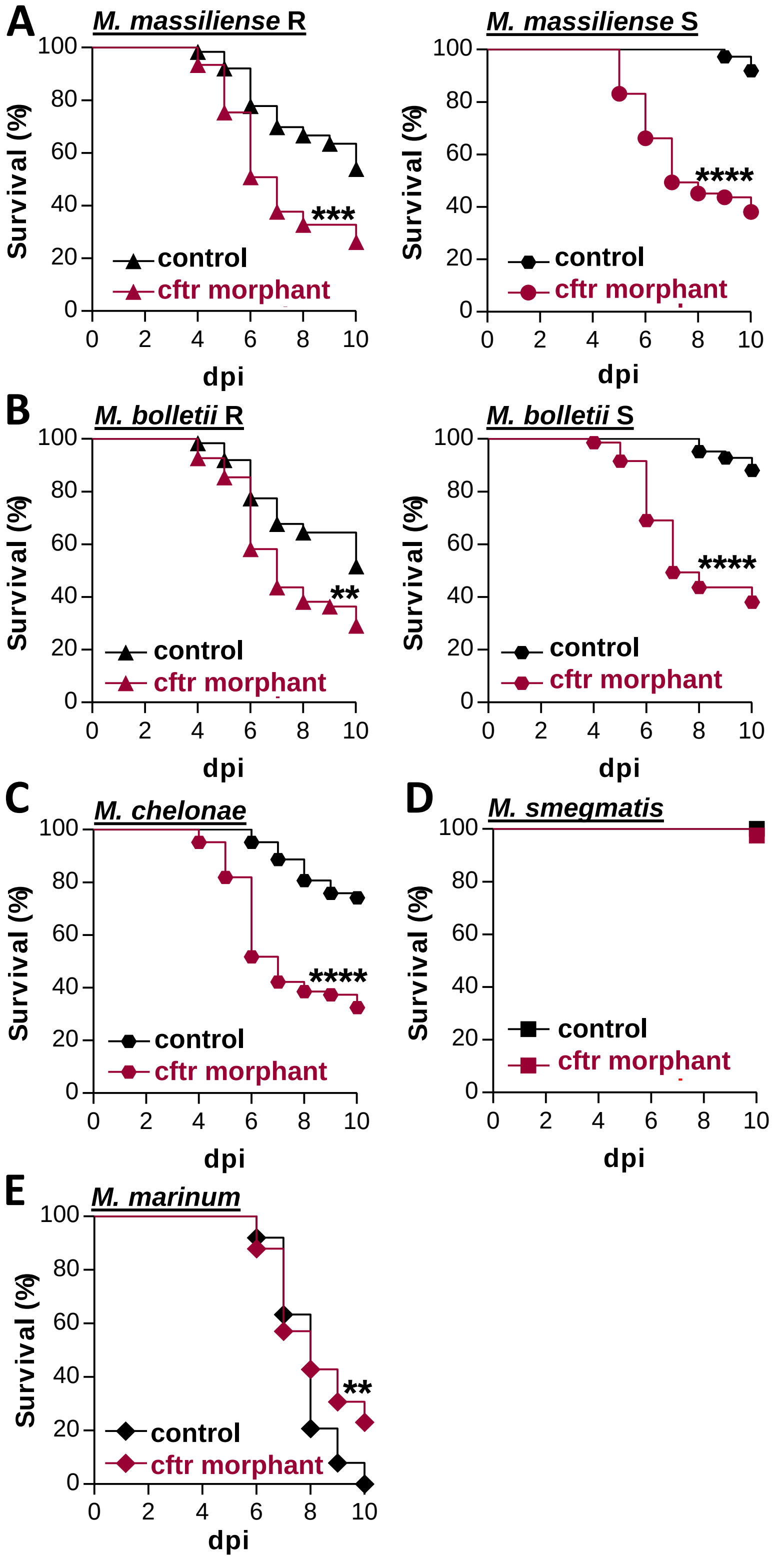

Figure S2. *Cftr* MO knockdown results in increased susceptibility to NTM, related to Figure 1

(A-E) Control embryos or *cftr* morphants were intravenously infected with ≈150 CFU of various mycobacteria expressing tdTomato. Data are plotted as percentage of surviving animal on each day (n=40, average of two independent experiments, Mantel-Cox Log-rank test).

(A) Survival analysis of embryos infected with either R (top graph) or S (bottom graph) variants of *M. massiliense*.

(B) Survival analysis of embryos infected with either R (top graph) or S (bottom graph) variants of *M. bolletii*.

(C) Survival analysis of *M. chelonae*-infected larvae.

(D) Survival analysis of *M. smegmatis*-infected larvae.

(E) Survival analysis of *M. marinum*-infected larvae.

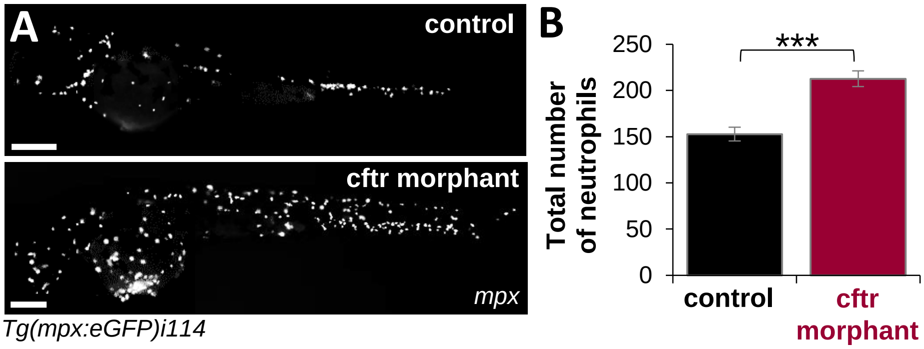

Figure S3. *cftr* knockdown promotes a basal hyperneutrophilia, related to Figure 4

(A) Representative fluorescence microscopy images of control embryos *versus cftr* morphants *Tg(mpx:eGFP)i114* embryos at 2 dpf. Scale bars, 200 µm.

(B) Basal number of neutrophils in whole embryos at 2 dpf. Graphs represent the mean ±SEM of two independent experiments (n=10). Significance was assessed by two-tailed unpaired Student's t test comparing both infected embryos per category.

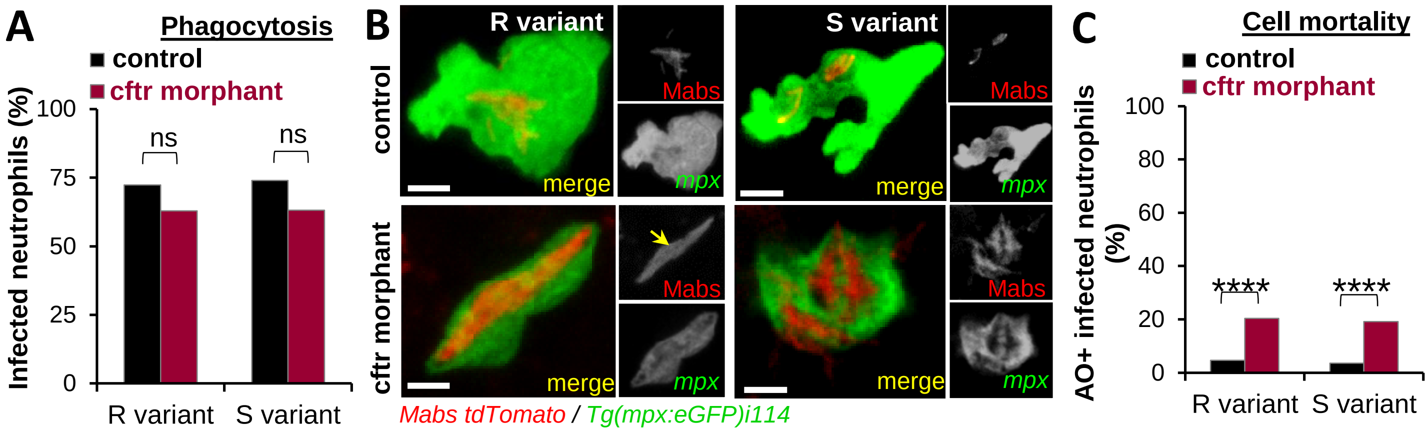

Figure S4, *cftr* knock-down inhibits *M. abscessus* killing inside neutrophils, related to Figure 4

(A) Control or *cftr* morphants *Tg(mpx:eGFP)i114* larvae were infected into the muscle with ≈100 CFU Mabs R or S expressing tdTomato. Proportion of infected neutrophils analyzed using confocal microscopy and recruited in the infected site at 4 hpi. Data are plotted as mean ± SEM from two independent experiments (n=20).

(B) Control or *cftr* morphants *Tg(mpx:eGFP)i114* embryos were *iv* infected with Mabs R or S expressing dtTomato. Confocal images showing infected neutrophils. While WT-neutrophils efficiently contain intracellular bacilli, CF-neutrophils appear overloaded with Mabs. Arrow indicates intracellular Mabs R cording. Scale bar, 2 µm.

(C) Control or *cftr* morphants *Tg(LysC_DSred)nz5* embryos were *iv* infected with ≈100 CFU Mabs R or S expressing E2-Crimson and stained for dead cells using acridine orange (AO). Proportion of dead infected neutrophils was evaluated in the CHT at 2 dpi using confocal microscopy. Data are plotted as mean ± SEM from two independent experiments (n=20).

Significance was assessed by Fisher's exact test (A and C).

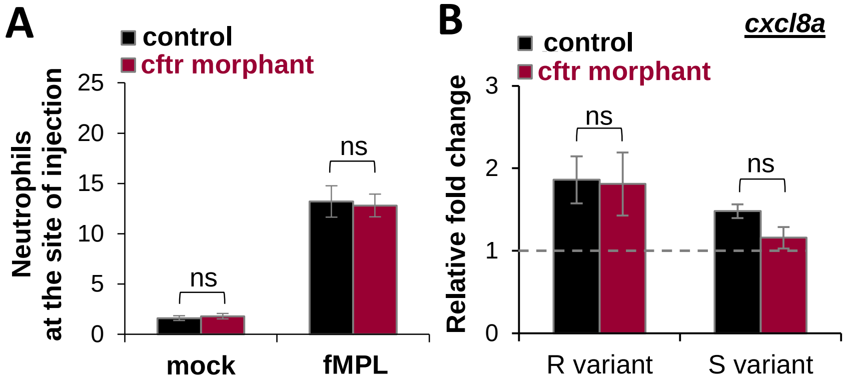

Figure S5. *cftr* knock-down does not impair the neutrophil chemotaxis functions, related to Figure 4

(A) Mean ± SEM number of recruited neutrophils into the otic cavity in response to mock or fMLP injection in control and *cftr* morphants *Tg(mpx:eGFP)i114* larvae monitored using confocal microscopy at 3 hpi (two independent experiments, n=14).

(B) qRT-PCR measurement for whole embryos 4 dpi after intravenous infection with ≈150CFU Mabs R or S and plotted as fold increase over mock injection for *cxcl8a*. Mean relative ± SEM gene expression of three independent replicates.

Significance was assessed by two-tailed unpaired Student's t test comparing both infected embryos per category (A and B).

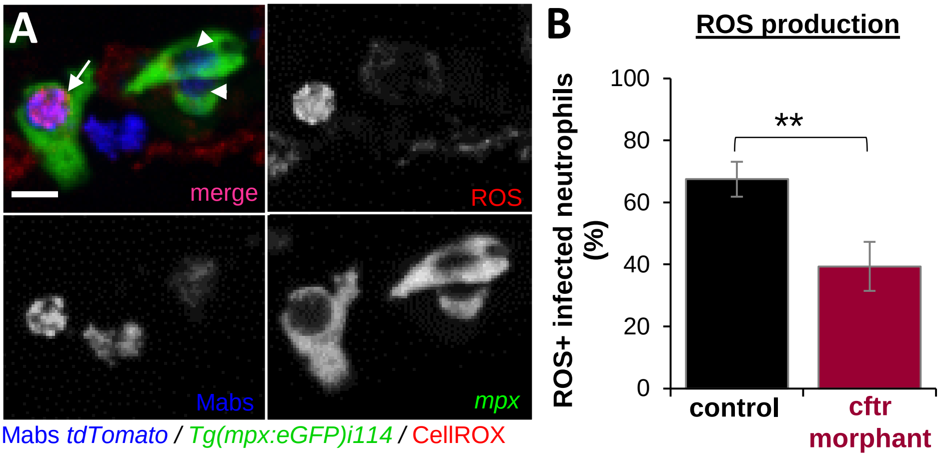

Figure S6, *cftr* knock-down altered neutrophil ROS production, related to Figure 6

(A-B) Control or *cftr* morphants *Tg(mpx:eGFP)i114* larvae were infected with ≈150 CFU Mabs expressing dtTomato into the muscle and stained for ROS production using CellRox Deep red.

(A) Confocal imaging showing both ROS^+ (arrow) or ROS^- (arrow heads) -producing infected neutrophils (arrow) at the infected site. Scale bar, 5 µm.

(B) Proportion of ROS-producing infected neutrophils were evaluated at 4 hpi using confocal microscopy (from two independent experiments (n=16)). Significance was assessed by Fisher's exact test.

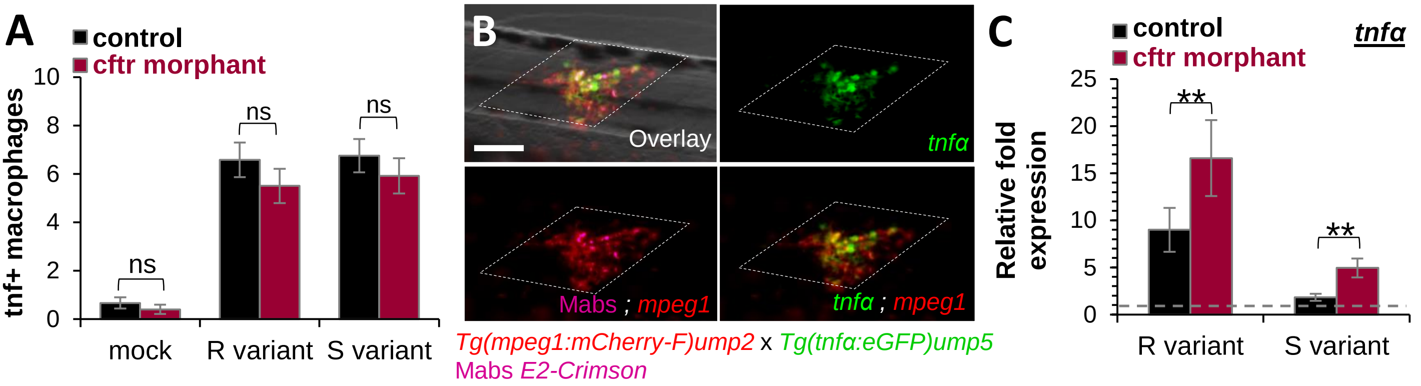

Figure S7. *M. abscessus* infection triggers hyperinflammation in the absence of CFTR, related to Figure 6

(A-B) Double transgenic larvae *Tg*(*tnfα*:*eGFP-F)ump5* / *Tg*(*mpeg1*:*mCherry-F*)*ump2)* were infected into the muscle with ≈100 Mabs R or S variants expressing E2-Crimson or with PBS (mock).

(A) Quantification of *tnf* $^{+}$ MΦ per infected larvae after 2 hpi. The data are representative of two experiments.

(B) Microscopy showing the representative expression of GFP^{+} cells at 2 hpi at the injection site. Scale bar, 70 µm.

(C) qRT-PCR measurement for whole Mabs R- or S-infected embryos 4 dpi (≈150 CFU) and plotted as fold increase over mock injection for *tnfα*. Mean relative ± SEM gene expression of three independent replicates. Significance was assessed by two-tailed unpaired Student's t test comparing both infected embryos per category (A and C).
